# Structure and replication cycle of a virus infecting climate-modulating alga *Emiliania huxleyi*

**DOI:** 10.1126/sciadv.adk1954

**Published:** 2024-04-10

**Authors:** Miroslav Homola, Carina R. Büttner, Tibor Füzik, Pavel Křepelka, Radka Holbová, Jiří Nováček, Marten L. Chaillet, Jakub Žák, Danyil Grybchuk, Friedrich Förster, William H. Wilson, Declan C. Schroeder, Pavel Plevka

**Affiliations:** ^1^ Central European Institute of Technology, Masaryk University, Brno, Czech Republic.; ^2^ Bijvoet Centre for Biomolecular Research, Utrecht University, Utrecht, Netherlands.; ^3^ Department of Botany and Zoology, Faculty of Science, Masaryk University, Brno, Czech Republic.; ^4^ Marine Biological Association, Plymouth, UK.; ^5^ School of Biological and Marine Sciences, University of Plymouth, Plymouth, UK.; ^6^ Veterinary Population Medicine, The University of Minnesota, St Paul, USA.

## Abstract

The globally distributed marine alga *Emiliania huxleyi* has cooling effect on the Earth’s climate. The population density of *E. huxleyi* is restricted by *Nucleocytoviricota* viruses, including *E. huxleyi* virus 201 (EhV-201). Despite the impact of *E. huxleyi* viruses on the climate, there is limited information about their structure and replication. Here, we show that the dsDNA genome inside the EhV-201 virion is protected by an inner membrane, capsid, and outer membrane. EhV-201 virions infect *E. huxleyi* by using fivefold vertices to bind to and fuse the virus’ inner membrane with the cell plasma membrane. Progeny virions assemble in the cytoplasm at the surface of endoplasmic reticulum–derived membrane segments. Genome packaging initiates synchronously with the capsid assembly and completes through an aperture in the forming capsid. The genome-filled capsids acquire an outer membrane by budding into intracellular vesicles. EhV-201 infection induces a loss of surface protective layers from *E. huxleyi* cells, which enables the continuous release of virions by exocytosis.

## Teaser

Cryo–electron microscopy was used to visualize cell entry and replication of a virus infecting climate-modulating alga *E. huxleyi*.

## INTRODUCTION

*Emiliania huxleyi* is a globally distributed single-celled marine alga known for its ability to multiply quickly in large ocean areas, resulting in blooms covering hundreds of thousands of square kilometers (*1*–*3*). Spherical *E. huxleyi* cells, with a diameter of 4 to 5 μm, are protected by calcite disks called coccoliths, which increase the albedo of seawater by reflecting light and thus decrease the amount of heat from solar radiation absorbed by oceans (*2*, *4*). This alga is an important component of the global carbon cycle, as the coccoliths shed from the cells descend to the sea bottom and serve as a sink for carbon dioxide (*5*). Furthermore, dimethyl sulfide and other compounds released by *E. huxleyi* promote the condensation of atmospheric aerosol droplets and the formation of clouds that reflect sunlight (*6*). These properties, in combination with broad distribution and high abundance, enable *E. huxleyi* to exert a cooling effect on the Earth’s climate (*2*, *7*, *8*).

*E. huxleyi* is susceptible to infection by *Nucleocytoviricota* viruses (NCVs), formerly commonly called nucleocytoplasmic large DNA viruses, which reduce the population density of the alga and alter its impact on the climate (*9*–*11*). NCVs infecting algae belong to the family Phycodnaviridae from the order Algavirales (*12*, *13*). *E. huxleyi* virus 201 (EhV-201) and closely related EhV-86 belong to the genus *Coccolithovirus* (*14*–*16*). More than 20 viruses from the family Phycodnaviridae that infect *E. huxleyi* have been isolated; however, only EhV-86 genome has been fully sequenced to date (*14*, *17*). The genome of EhV-86 has a size of 407 kilo–base pair with 472 predicted protein-coding sequences (*14*).

Virions of coccolithoviruses are unique within the family Phycodnaviridae because they contain not only a membrane inside the capsid but also an additional membrane wrapped around the outer capsid surface, similar to the African swine fever virus from the family Asfaviridae (*18*–*21*). It has been speculated that EhV-86 delivers its genome into cells by the fusion of its outer membrane with a cell membrane, similar to enveloped animal viruses (*19*). Furthermore, it has been proposed that the capsid of EhV-86 enters the cytoplasm intact and releases its genome into the nucleus (*19*). EhV-86, similar to other phycodnaviruses, probably replicates its genome in the cell nucleus, but progeny particles assemble in the cytoplasm (*19*). Toward the end of the virus replication cycle, which takes ~5 hours, the cytoplasm of *E. huxleyi* cells contains tens of progeny virions (*19*). It has been speculated that EhVs acquire their outer membrane by budding from the host cell membrane (*18*, *19*). However, there is evidence of accumulation of the newly formed EhV virions in intracellular vesicles (*22*, *23*). Despite previous studies of EhVs and possible analogies with the related better-studied *Paramecium bursaria* chlorella virus 1 (PBCV-1) (*24*–*26*), many aspects of EhV structure and replication remain unclear.

Here, we used various electron microscopy approaches to show that the EhV-201 infection process is different than previously inferred based on data obtained using lower-resolution techniques. The EhV-201 virion delivers its genome into the algal cytoplasm by fusing its inner membrane with the plasma membrane. The capsid, together with the outer membrane, remains attached to the cell surface. After genome replication in the nucleus, EhV-201 capsid assembly initiates in the cytoplasm synchronously with genome packaging on membrane segments derived from the endoplasmic reticulum. Upon completion of the genome packaging, EhV-201 particles bud into intracellular vesicles and thus acquire their outer membrane. EhV-201 infection induces the loss of surface protective layers from *E. huxleyi* cells, which enables the continuous release of progeny virions by exocytosis.

## RESULTS AND DISCUSSION

### Structure of EhV-201 virion

**Fig. 1.**
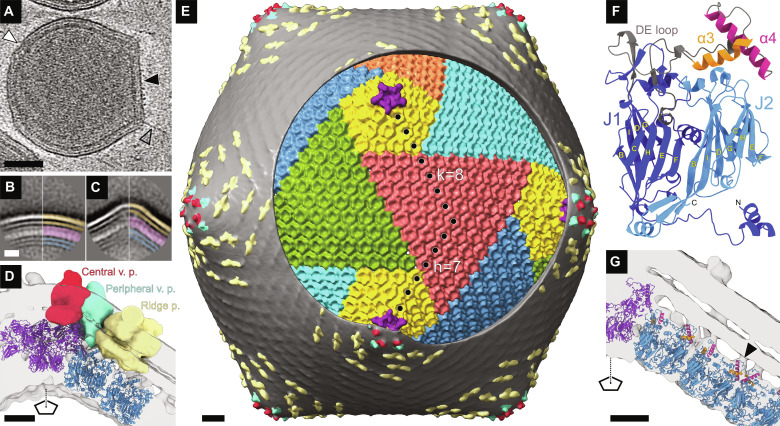
Structure of EhV-201 virion. (**A**) Central section from cryo-tomogram of EhV-201 virion. The part of the particle exhibiting icosahedral arrangement is indicated by a black arrowhead, the deformed part by a white arrowhead, and a filament protruding from a virion vertex by a transparent arrowhead with a black outline. Scale bar, 50 nm. (**B** and **C**) Reference-free two-dimensional (2D) class averages of round (B) (*N* = 922) and angular (C) (*N* = 1012) segments of EhV-201 virions. The inner membrane is highlighted in blue, the capsid in magenta, and the outer membrane in orange. Scale bar, 10 nm. (**D**) Cross section of angular virion vertex. Transmembrane proteins are shown in surface representation and distinguished by colors: One copy of the central vertex protein (v. p.) is shown in red, one copy of the peripheral vertex protein (v. p.) in light blue, and dimer of ridge proteins (p.) in light yellow. The capsid proteins are shown in cartoon representation: penton protein in purple and major capsid proteins in blue. Scale bar, 5 nm. (**E**) Composite map of EhV-201 virion. The surface of the virion is covered with the outer membrane (gray) with central (red) and peripheral (light blue) vertex proteins and dimers of ridge proteins (light yellow and gray ripples on the virion surface). A circular region of the outer membrane was removed. Penton proteins are shown in purple whereas major capsid proteins are in various other colors. Scale bar, 10 nm. (**F**) AlphaFold2-predicted structure of EhV-201 major capsid protein with double jelly roll fold. (**G**) Cross section of EhV-201 virion vertex showing interactions of amphipathic helices α3 (orange) and α4 (magenta) from J1 domain of major capsid proteins with outer virion membrane (an example is indicated by a black arrowhead). The penton proteins are shown in purple and major capsid proteins in blue. Scale bar, 5 nm.

Virions of EhV-201 are spherical in shape with a maximum outer diameter of 211 nm (Fig. 1A and fig. S1). The EhV-201 genome is protected by a 4.2-nm-thick inner membrane, a 6.1-nm-thick capsid, and a 6.1-nm-thick outer membrane (Fig. 1, A to C, and fig. S2). Unlike virions of NCVs with isometric capsids that have been structurally characterized to date (*20*, *21*, *24*, *26*–*34*), those of EhV-201 are deformed, and parts of their capsids lack angular icosahedral features (Fig. 1A and fig. S1). We used sub-tomogram averaging to reconstruct a vertex with regular features and expanded the map according to icosahedral symmetry to obtain a complete EhV-201 virion structure with a resolution of 18 Å (Fig. 1, D and E, fig. S3, A to C, and table S1). The virion reconstruction enabled the identification of three types of transmembrane proteins embedded in the outer membrane: (i) Five copies of the central vertex transmembrane protein (indicated by the red density in Fig. 1, D and E, and fig. S4) are located around each fivefold symmetry axis and bind to pentamers of penton capsid protein. (ii) Five copies of the peripheral vertex transmembrane protein (indicated by the light blue density in Fig. 1, D and E, and fig. S4), which are positioned around the five central vertex proteins, bind to pentamers of the penton capsid protein (Fig. 1, D and E, and fig. S4). (iii) Elongated ridges that cover most of the EhV-201 virion surface are formed by dimers of ridge transmembrane proteins (indicated by the yellow density in Fig. 1, D and E, and fig. S4). Each dimer of ridge proteins binds to two underlying hexamers of major capsid proteins (Fig. 1, D and E, fig. S4). The EhV-201 virion contains 60 copies of each central and peripheral vertex protein and at least 1320 copies of ridge protein (Fig. 1E).

The capsid of the mature EhV-201 virion has a maximum diameter of 199 nm and a triangulation number of 169 (*h* = 7, *k* = 8) (Fig. 1E and fig. S5). Capsomers of major capsid proteins (YP_293839, locus EhV085) are organized into penta-symmetrons and tri-symmetrons, as in other NCVs (Fig. 1E and figs. S6, A to C, and S7) (*35*). The structure of the EhV-201 major capsid protein, predicted using AlphaFold2 (*36*), has the characteristic double jellyroll fold of the capsid proteins of NCVs and other viruses (Fig. 1F and fig. S6A) (*35*). The jellyroll cores J1 and J2 are each composed of two β sheets named according to the convention BIDG and CHEF (Fig. 1F) (*35*). Three copies of the major capsid protein form a capsomer with quasi-sixfold symmetry (fig. S6, A to C) [map calculated from the predicted capsid protein structure fits into the corresponding part of the cryo–electron microscopy (cryo-EM) map with the correlation coefficient of 0.886]. Pentamers of penton protein (YP_293955, locus EhV201) are positioned around icosahedral fivefold axes (Fig. 1E and fig. S7). The AlphaFold2-predicted and modeled structure of the EhV-86 penton protein can be divided into a single jellyroll domain and a large 538-residue flower-like “insertion domain,” which is composed of five small globular domains (fig. S6, D to F, and table S2). A pentamer of penton protein fits well into the cryo-EM reconstruction (CC = 0.887 using a map calculated from the protein structure), demonstrating its interaction with the central and peripheral vertex proteins (Fig. 1D and fig. S4). The outer surface of the pentamer is positively charged, which may enable its interactions with the vertex proteins and the outer virion membrane (fig. S6, G to I).

The EhV-201 inner virion membrane is less well resolved than the outer one, indicating higher variability in its structure between individual particles (Fig. 1, A to C, and fig. S8). The reconstruction, because of its limited resolution, did not enable identification of minor capsid proteins mediating contacts between the inner membrane and the capsid, as is the case in other NCVs such as Tokyovirus, PBCV-1, and African swine fever virus (*20*, *21*, *24*, *26*, *34*).

### Interactions between the outer membrane and capsid

The transmembrane proteins from the EhV-201 outer membrane bind to the capsid; however, there are additional interactions between the major capsid proteins and the outer membrane. The predicted structure of major capsid protein of EhV-201 contains a 96-residue-long loop between β strands D and E of the J1 jellyroll domain (Fig. 1F and fig. S6A). The loop is predicted to form helices α3 and α4, which are 13- and 20-residue long, respectively, and are positioned at the outer surface of the capsid (Fig. 1, F and G). Helices α3 and α4 contain hydrophobic residues organized in a moderate amphipathic α-helical arrangement, which indicates that they may bind membranes (fig. S9). Fitting the predicted EhV-201 capsomer structure into the sub-tomogram reconstruction of the virion vertex positions helices α3 and α4 inside densities connecting the capsid to the outer membrane (Fig. 1G). Therefore, we speculate that the amphipathic helices attach the outer virion membrane to the capsid. The abundant capsid-outer membrane contacts may enable EhV-201 virions to withstand deformation without negatively affecting the infectivity of the virus.

### Filaments protruding from vertices of EhV-201 virions

Tomograms of 96% of EhV-201 virions (*N* = 50) contained at least one 3.3-nm-thick (SD = 0.5, *N* = 20) and 30- to 150-nm-long (mean = 72, SD = 31, *N* = 20) filament protruding from a fivefold particle vertex (Fig. 1A and fig. S10). The filaments emerge from the outer membrane, but their exclusive positioning at the vertices provides evidence that they bind to specific sites at the capsid (Fig. 1A and fig. S10). We identified particles containing more than one such filament, indicating that it is unlikely that the filament is a feature of a special vertex in the EhV-201 virion (fig. S10). The classification of sub-tomograms of EhV-201 virion vertices did not identify a subpopulation of vertices containing the putative filament. The filament may be a feature that is too weak and flexible to be detected by the classification procedure. This analysis also did not provide any evidence of the existence of a special vertex in EhV-201 virions. The putative function of EhV-201 filaments in *E. huxleyi* cell infection may be similar to filaments of PBCV-1, virions of which have been observed to attach to cell walls via hair-like fibers (*37*). However, unlike the putative fibers of EhV-201, those of PBCV-1 are anchored at the faces of icosahedral capsids and may be of several types (*26*, *37*). There is evidence of specific protein receptor-ligand interactions during initial EhV attachment engagement (*38*), which may be mediated by the putative fibers. Furthermore, EhV-201 virions might contain internal fibers (fig. S10). Internal fibers with a diameter of about 6 nm were previously observed using atomic force microscopy in mechanically disrupted virions of Mimivirus (*30*).

### EhV-201 attachment and genome delivery

Many NCVs characterized to date deliver their genomes into the host cell cytoplasm by fusing their inner capsid membrane with the host plasma membrane (*24*, *39*–*42*). This mechanism of genome delivery is characterized by the capsid and emptied inner membrane sack remaining attached to the surface of an infected cell (*24*, *41*). In contrast, it has been speculated that EhV-86 infects cells via the fusion of its outer virion membrane with the plasma or endosome membrane, which would result in the delivery of the genome enclosed within the inner membrane and capsid into the host cytoplasm (*18*).

**Fig. 2.**
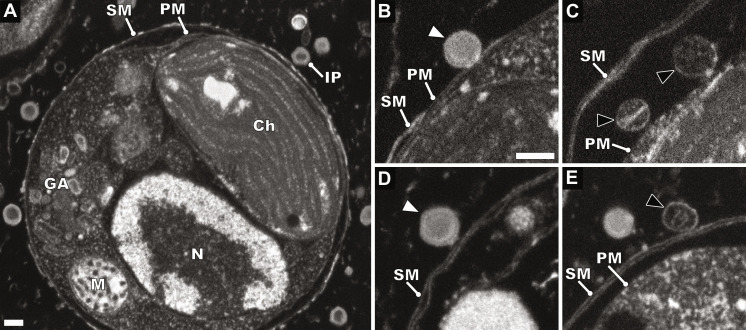
Attachment and genome delivery of EhV-201. (**A**) Scanning electron micrograph of high-pressure vitrified and resin-embedded *E. huxleyi* cell infected by EhV-201 at multiplicity of infection (MOI) = 10, 30 min after infection. IP, infecting particle; Ch, chloroplast; GA, Golgi apparatus; M, mitochondrion; N, nucleus; SM, surface membrane; and PM, plasma membrane. Scale bar, 200 nm. (**B**) Genome-containing EhV-201 particle (white arrowhead) attached to plasma membrane of cell. (**C**) Empty particles (black arrowheads with white outlines) are attached to plasma membrane of cell. (**D**) Genome-containing EhV-201 particle attached to surface membrane of *E. huxleyi* cell. (**E**) EhV-201 particle that abortively released its genome after binding to surface membrane (black arrowhead with white outline). Scale bar (B to E), 200 nm.

**Fig. 3.**
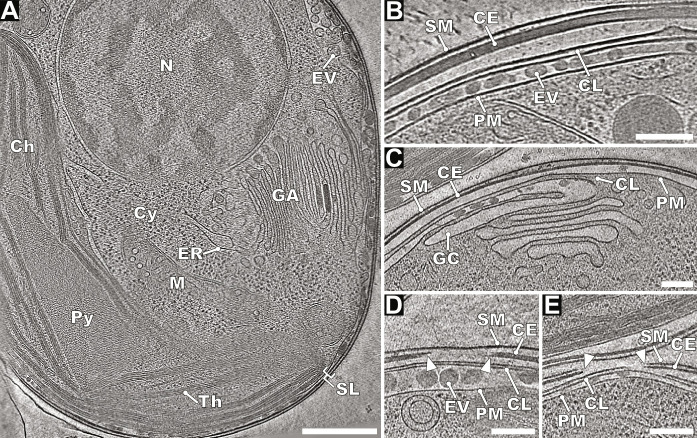
Native structure of *E. huxleyi* cell. (**A**) Projection image of 30-nm-thick section of cryo-tomogram of noninfected *E. huxleyi* cell from non-calcifying strain CCMP 2090. N, nucleus; Cy, cytoplasm; Ch, chloroplast; Th, thylakoid stacks; Py, pyrenoid; ER, endoplasmic reticulum; GA, Golgi apparatus; M, mitochondrion; and SL, surface layers. Scale bar, 500 nm. (**B** to **E**) Details of organization of protective layers at *E. huxleyi* surface. (B) Detail of continuous cell surface layers. Extracellular vesicle (EV) between the plasma membrane (PM) and cytoplasmic leaflet (CL), cell envelope (CE), and surface membrane (SM). (C) Cytoplasmic leaflet probably originates from fusion of GC Golgi apparatus cisternae with plasma membrane. (D) Opening in cell envelope, indicated by white arrowheads, is covered with a surface membrane. (E) Opening in both the surface membrane and cell envelope makes plasma membrane accessible for virus infection. Scale bars, (B to E) 200 nm.

We used serial block-face scanning electron microscopy of vitrified and resin-embedded *E. huxleyi* cells to observe EhV-201 attachment and genome delivery (Fig. 2, fig. S11, and movie S1). To facilitate the preparation of samples for electron microscopy studies, we used *E. huxleyi* strain CCMP 2090, which does not produce coccoliths (Figs. 2 and 3 and fig. S12A) (*43*). In most cases, EhV-201 particles attached to *E. huxleyi* cells were oriented with one of their fivefold vertices toward the cell surface (Fig. 2, B and C, and fig. S11). We never observed capsids of EhV-201 entering cells. Some of the EhV-201 particles attached to the *E. huxleyi* surface contained emptied and partly collapsed inner membranes (Fig. 2, C and E, and fig. S11, G to I), indicating that they released their genomes by fusion of the inner virus membrane with the plasma membrane. This type of genome delivery requires the opening of the outer membrane and capsid of EhV-201. We hypothesize that the binding of the central and peripheral vertex proteins or of the putative filament to the host cell may trigger the conformational changes required for the outer membrane and capsid opening. Thus, the capsid enveloped by the outer membrane may remain associated with the plasma membrane, as observed in images of the infected *E. huxleyi* cells (Fig. 2, B and C, and fig. S11). Genome delivery using the fusion of inner capid membrane with cytoplasmic membrane has been previously described for PBCV-1 (*24*), and fusion of the inner capsid membrane with endosome membrane has been demonstrated for African swine fever virus (*44*).

### Cell surface layers of *E. huxleyi*

Most of the surface of *E. huxleyi* is covered with coccoliths, which, nevertheless, were shown to provide only limited protection against EhV infection (*45*). We used focused ion beam milling (FIBM) and cryo–electron tomography (cryo-ET) to show that EhV-201 virions can diffuse through the spaces in the coccolith structure and openings in the other surface layers to reach the plasma membrane (fig. S13). Except for the missing coccoliths, CCMP 2090 cells are covered with the same surface layers that are found in wild-type cells: the surface membrane, cell envelope formed of polysaccharides, and cytoplasmic leaflets, large flat folds of plasma membrane that wrap around the cell surface (Fig. 3, fig. S14, and movie S2) (*46*). Cell envelope thickness ranges among the cells from 22 to 62 nm (mean = 32 nm, SD = 13, *N* = 10), whereas the cell envelope of one cell is uniform in thickness (SD < 10%) (fig. S15). We observed several EhV-201 particles that attached to or fused their inner viral membranes with the cell surface membrane, which resulted in the abortive release of the virus genomes into the extracellular space (Fig. 2, D and E, and fig. S11, F to I). This finding suggests that the surface membrane protects *E. huxleyi* cells from EhV infection. Furthermore, the cell envelope is impenetrable to virus particles. However, the cell envelopes were not resolved in the electron micrograph of resin-embedded cells (Fig. 2 and fig. S14). We speculate that the cell polysaccharide envelope could not be detected because it was not stained by the osmium tetraoxide and uranyl acetate used for sample contrasting (*47*, *48*), or it could have been dissolved by the sample preparation procedure (*49*). The cryo-ET reconstructions of control *E. huxleyi* cells demonstrate that surface membranes and cell envelopes contain openings a few hundred nanometers in diameter (Fig. 3, C and D, and fig. S13). It is likely that the exposed areas of the plasma membrane render *E. huxleyi* cells sensitive to infection. The differences in the extent of cell coverage and, thus, in the protection provided by the surface membrane and envelope to individual *E. huxleyi* cells make the EhV–*E. huxleyi* interaction complex at the population level. EhV-201 infection at a multiplicity of infection (MOI) of 10 did not clear the affected *E. huxleyi* culture after one virus replication cycle (fig. S12B). Only 1.4% (*N* = 211) and 19.4% (*N* = 227) of *E. huxleyi* cells had EhV-201 particles attached to their surface when infected with MOIs of 10 and 100, respectively (figs. S12C and S16). Our results agree with previous observations demonstrating that at most 25% of *E. huxleyi* cells from a population display infection symptoms at a given time (*37*, *50*–*52*).

### EhV-201 genome replication

**Fig. 4.**
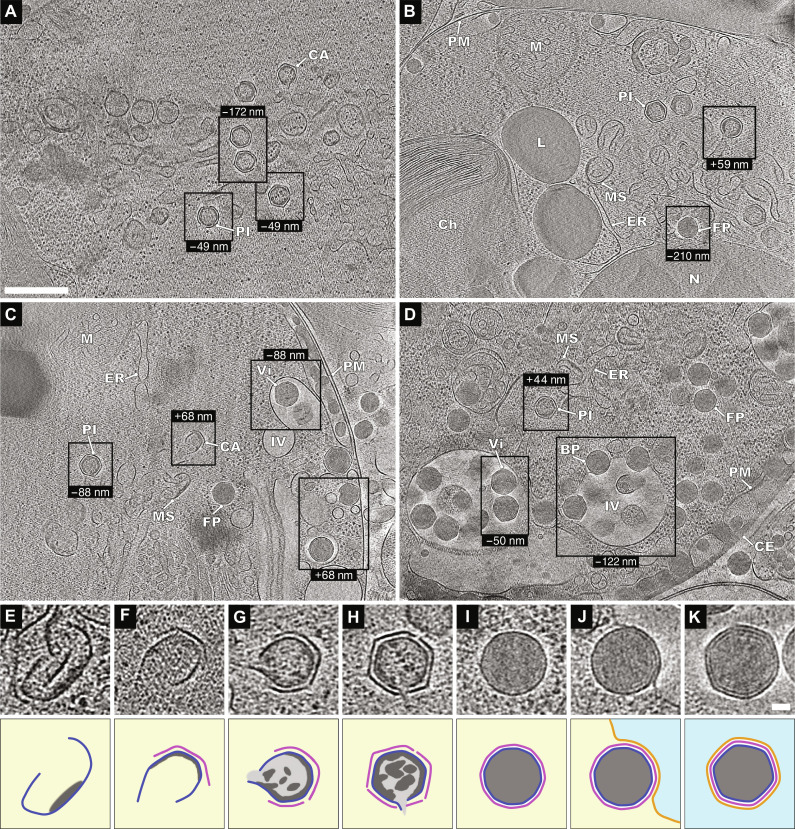
EhV-201 factories in *E. huxleyi* cells and EhV-201 virion assembly pathway. (**A** to **C**) Virus factories in cells that lost surface protective layers and could continuously release virions by exocytosis (80% of cells). (**D**) Virus factory with accumulated virions in cell with intact surface layers (20% of cells). The panels show projection images of 30-nm-thick tomogram sections of infected *E. huxleyi* cells. N, nucleus; Ch, chloroplast; M, mitochondrion; ER, endoplasmic reticulum; L, lipid droplet; PM, plasma membrane; and CE, cell envelope. Components of EhV-201 factories: MS, membrane segments; CA, capsid assembly intermediate; PI, genome packaging intermediate; FP, full particle; BP, budding particle; Vi, virion; and IV, internal vesicle. (A) Interior of a cell, (B to D) regions of cells from center to cell periphery indicated by a plasma membrane or cell envelope. Scale bars, (A to D) 500 nm. (**E** to **K**) Assembly stages of EhV-201 particles with corresponding schematic drawings underneath. (E) Membrane segment (blue) with associated density that may correspond to condensing virus genome (dark gray). The yellow background indicates cytoplasm. (F) Capsid (magenta) assembly is initiated at outer surface of membrane segment. (G) Early genome packaging intermediate with incomplete capsid and membrane sack. The genome is indicated by light and dark gray. (H) Genome packaging intermediate with inner membrane sack containing a single opening for DNA entry. Several openings in the capsid can be distinguished. The capsid has straight edges and angular vertices, indicating quasi-icosahedral symmetry. (I) A particle containing a complete genome. The capsid has become more oblique than that of the genome packaging intermediate, and the genome is homogeneously distributed. (J) Budding of full particles into an intracellular vesicle. The membrane of the vesicle is indicated by an orange line, and its interior by light blue. (K) Virion inside an intracellular vesicle. Scale bars, (E to K) 50 nm.

After delivery into the cytoplasm, the genomes released from EhV-201 particles cannot be identified in images obtained using either serial block-face scanning electron microscopy or in cryo-tomograms of infected *E. huxleyi* cells. However, EhV-201 infection induces changes in the internal organization of the infected cells that enable indirect identification of the virus replication sites. Some NCVs, including Mimivirus and poxviruses, replicate their genomes in dense cytoplasmic replication factories (*53*, *54*). In contrast, the remaining NCVs replicate in the cell nucleus, and their virus factories, which serve only for virion assembly, do not contain dense areas. Cryo-tomograms of EhV-201–infected *E. huxleyi* cells do not contain dense cytoplasmic virus factories, which corroborates previous evidence that the cocolithoviruses replicate in cell nuclei (Fig. 4, A to D) (*18*, *19*). Whereas the nuclei of noninfected *E. huxleyi* cells contain distinct regions of heterochromatin and euchromatin and are enveloped by two membranes (*N* = 35) (Fig. 3A), the nucleus of an infected cell is characterized by a uniform distribution of its content and the absence of the outer nuclear membrane (Fig. 4B). Similar changes in the nuclear structure were reported previously for infections by NCVs that replicate in cell nuclei (*55*, *56*).

### Disruption of endoplasmic reticulum and outer nuclear membrane by EhV-201 infection

The assembly of EhV-201 particles occurs in viral factories that occupy a segment of the cell cytoplasm between the nucleus and plasma membrane that is devoid of normal cellular organelles (Fig. 4, A to D). The viral factories are surrounded by nucleus, chloroplast, mitochondria, and lipid droplets. EhV-201–infected *E. huxleyi* cells do not contain the characteristic endoplasmic reticulum, and their outer nuclear membranes are also partially or completely disrupted (Fig. 4, A to D). We speculate that EhV-201 infection induces the disintegration of the endoplasmic reticulum and outer nuclear membranes into segments that are the most abundant components of the viral factories (Fig. 4, A to D). The edges of the membrane segments are thermodynamically unfavorable structures that have to be stabilized by special proteins and lipids, the synthesis of which is probably ensured by EhV-201 (*14*, *55*, *57*–*59*).

### EhV-201 capsid assembly and genome packaging

EhV-201 virion assembly initiates at a surface of a membrane segment located in the virus factory (Fig. 4, E to K). The early assembly intermediate consists of a membrane segment lined on one side by a featureless electron-dense layer (Fig. 4E), which has a similar appearance to that of incompletely packaged genomes inside assembling particles, and may therefore correspond to the initial stages of genome condensation (Fig. 4, F and G). Alternatively, the electron-dense layer could represent scaffold proteins mediating the bending of the membrane segment. The face of the membrane segment opposite to that associated with the electron-dense layer serves as a nucleation site for capsid assembly (Fig. 4, F and G). The forming capsids have straight edges and angular vertices, which indicate that they assemble according to the rules of quasi-icosahedral symmetry as is the case for NCVs with isometric capsids (Fig. 4, G and H) (*27*, *30*, *35*, *39*, *40*, *55*). An assembling capsid gradually encloses the membrane segment it is associated with and, in the process, induces its bending into a membrane sack (Fig. 4, G and H). As the capsid assembly progresses, the virus DNA is packaged through an aperture in the forming capsid and the underlying membrane (Fig. 4H). When the assembly of the capsid nears its completion, the diameter of the DNA-packaging aperture in the capsid and membrane sack decreases to 15 to 40 nm (mean = 28, SD = 8, *N* = 6) (Fig. 4H). The capsid of EhV-201 can have several openings; however, we observed at most one aperture in the inner capsid membrane that served for genome packaging (Fig. 4H). Therefore, we speculate that genome can be packed into a given particle only through one opening. During packaging, the EhV-201 genome forms condensed clusters inside the membrane sack (Fig. 4, F to H). The mechanism of genome packaging of EhV-201, and, probably, also of other NCVs, is distinct from that of tailed bacteriophages and herpesviruses, in which the double-stranded DNA is pumped into the preformed capsid through a protein portal complex (*60*, *61*).

### EhV-201 budding into intracellular vesicles and outer membrane acquisition

The EhV-201 particles in the genome-filling stage are characterized by (i) angular capsids with a diameter of 193 nm (SD = 4), (ii) inner membranes separated from capsids, and (iii) clusters of packaged DNA (Fig. 4, F to H, and fig. S12D). When the genome packaging completes, the capsid becomes more spherical with a diameter of 190 nm (SD = 2) (Fig. 4I and fig. S12D), the inner membrane sack adheres to the capsid, and the genome distribution changes to homogeneous (Fig. 4I). Whereas the genome packaging intermediates exhibit no affinity for membranes, the genome-filled round particles bud into intracellular vesicles (Fig. 4J). Therefore, we speculate that the change in the capsid shape is connected to conformational changes in the major capsid proteins, which expose their amphipathic helices α3 and α4 from the DE loop of the J1 domain at the particle surface and thus enable binding to membranes. This conformational change may also enable the binding of the capsid to EhV-201 transmembrane proteins, which must be present in the vesicles that the particles bud into. The budding of EhV-201 into vesicles is probably driven by the high-avidity interactions between the capsid and the vesicle membrane. The virions that completed budding and acquired an outer membrane have a diameter of 210 nm (SD = 4) (Fig. 4K and fig. S12D). Previous observations of EhV-201 and EhV-86 inside vacuoles support our hypothesis of EhV-201 budding into intracellular vesicles (*22*, *23*). The budding process produces mature virions that need to be released from cells to initiate the next round of infection (Fig. 4D).

### Exocytosis of EhV-201 virions

**Fig. 5.**
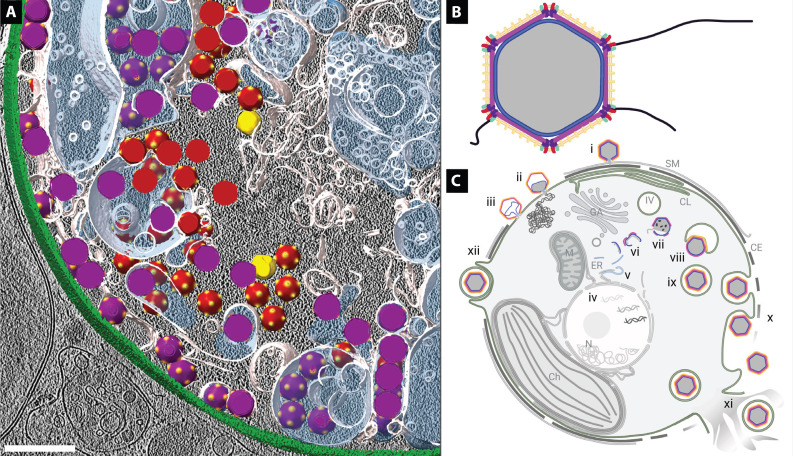
EhV-201 structure and replication. (**A**) 3D representation of cryo-tomogram of an EhV-201–infected cell. The cell envelope is shown in green, cellular membranes in white, the content of intracellular vesicles in semi-transparent blue, assembly intermediates in yellow, full particles in red, and virions in purple. Scale bar, 500 nm. (**B**) EhV-201 virion structure. The genome is shown in gray, the inner membrane in blue, the capsid in magenta, penton proteins in purple, the outer membrane in light orange, central vertex proteins in red, peripheral vertex proteins in light blue, the ridge proteins in yellow, and fibers in black. (**C**) Infection cycle of EhV-201. Abortive infection: (i) Surface membrane (SM) and cell envelope (CE) with underlying cytoplasmic leaflets (CL) protect *E. huxleyi* from EhV-201 infection; the viral genome is delivered into the extra-cytoplasmic space. Productive infection: (ii) EhV-201 virion fuses its inner membrane with plasma membrane to deliver its genome into the cytoplasm. (iii) Empty particle remains attached to the cell surface. (iv) EhV-201 genome replicates in the cell nucleus (N). (v) Segmentation of the endoplasmic reticulum (ER) and outer nuclear membrane. (vi) Genome packaging and capsid assembly initiate on opposite surfaces of membrane segments. (vii) Genome is packaged into a particle through an aperture. (viii) The completion of the genome packaging induces a conformational change in the capsid, which enables it to bud into intracellular vesicles (IV). (ix) Virion inside an intracellular vesicle. (x) EhV-201 infection causes the loss of surface protective layers from *E. huxleyi* cells, which enables the continuous release of virions by exocytosis. (xi) The EhV-201 replication cycle is terminated by cell lysis, which results in the release of virions inside vesicles. Alternative infection pathway: (xii) Phagocytosis of EhV-201 virions inside vesicles. Ch, chloroplast; M, mitochondrion; GA, Golgi apparatus. Panels (B) and (C) were created using BioRender.com.

The formation of EhV-201 virions by budding into intracellular vesicles predisposes them to their release from the infected cells by exocytosis. However, the cell envelope and surface cell membrane cover 93% (*N* = 43) of native *E. huxleyi* cells and could block the diffusion of virus particles from the cell surface (Fig. 3). Efficient virion release is possible because EhV-201 infection induces the loss of surface membrane, envelope, and cytoplasmic leaflets from 80% (*N* = 35) of *E. huxleyi* cells (Fig. 4, A to C). In the subset of infected *E. huxleyi* cells that retained their surface membrane and envelope, EhV-201 virions accumulated beneath the protective layers (Figs. 4D and 5A and movie S3). The budding into intracellular vesicles enables the continuous production and release of EhV-201 particles from the infected algae without the need for cell lysis. Previous studies by Mackinder *et al.* (*18*) and Schatz *et al.* (*19*) indicated that EhVs are released from cells by budding into the plasma membrane, based on electron microscopy images of thin sections of EhV-infected *E. huxleyi* cells. However, the technique did not enable imaging of the cell envelope, and the sample preparation induced a shrinkage of cellular structures. It is therefore possible that the images presented by Mackinder *et al.* (*18*) and Schatz *et al.* (*19*) did not show virus budding but instead corresponded to exocytosed particles trapped under the cell envelope, which may be more common in *E. huxleyi* cells covered by coccoliths. Maturation processes involving budding into internal vesicles have been described for other enveloped NCV families that infect animals and amebas, including Asfaviridae, Poxviridae, and Mimiviridae (*55*).

### Vesicle-embedded EhV-201 virions

The EhV-201 infection of *E. huxleyi* is terminated by cell lysis (*10*, *18*), which causes the release of virions embedded in vesicles (fig. S17). We never observed infection of *E. huxleyi* by vesicle-bound EhV-201 particles; nevertheless, it has been shown that *E. huxleyi* cells in the late stationary phase phagocytose particles with a diameter of up to 500 nm (*62*). Therefore, the vesicle-bound virions may be phagocytosed by the alga, which could result in infection, as has been suggested by Mackinder *et al.* (*18*). Alternatively, the vesicle-bound particles could become infectious after the disruption of the vesicle membrane.

### EhV-201 structure and infection cycle

The EhV-201 virion initiates infection by binding to a cellular membrane using a particle vertex (Fig. 5, B and C). Our results indicate that an EhV-201 particle delivers its genome into the cytoplasm by fusing its inner membrane with the plasma membrane of a cell (Fig. 5C). Attachment to the plasma membrane and opening of the EhV-201 capsid is probably mediated by transmembrane proteins positioned around the fivefold vertex of the particle or by a filament protruding from the vertex (Fig. 5B). Most *E. huxleyi* cells at a given time are resistant to infection by EhVs (*37*, *50*–*52*), probably because of the protection provided by the surface membrane and cell envelope that restrict access of the virus particles to the plasma membrane. The absence of dense structures in the cytoplasm of EhV-201–infected cells indicates that the virus genome replicates in the cell nucleus (Fig. 5, A and C, and movie S3). The progeny particles assemble in virus factories located in the cytoplasm (Fig. 5, A and C). Capsid assembly is initiated at the surface of endoplasmic reticulum–derived membrane segments (Fig. 5, A and C). The genomes are packaged into the forming capsids through large apertures in the capsid and underlying membrane. After completion of the genome packaging, the capsids change their conformation, which enables them to acquire an outer membrane by budding into intracellular vesicles (Fig. 5, A and C). EhV-201 infection induces the loss of the surface membrane and cell envelope from *E. huxleyi* cells, which enables the continuous release of EhV-201 virions by exocytosis (Fig. 5C). The EhV-201 replication cycle is terminated by cell lysis, which results in the release of virions inside vesicles from the ruptured cells (Fig. 5C). The vesicle-embedded virions can initiate infection if phagocytosed by *E. huxleyi* or after disruption of the vesicle membrane (Fig. 5C). Our study opens up numerous questions related to the molecular mechanisms of specific steps of the EhV-201 replication cycle such as: (i) What are the molecular interactions that enable the attachment of EhV-201 to the cellular membranes? (ii) How does the EhV-201 particle open to enable genome delivery? (iii) How does EhV-201 infection induce the loss of surface membrane and cell envelope to facilitate continuous virion release?

Characterization of the EhV-201 replication cycle contributes to our understanding of the general replication strategies used by NCVs and highlights similarities in the nature of genome delivery and particle assembly across different viral families within the NCV group. *E. huxleyi* is a globally abundant marine phytoplankton species, and viral infections affect its population dynamics. Understanding the infection cycle of EhV-201, including its attachment, replication, and release mechanisms, can help to explain the dynamics of viral infections in marine environments and their potential consequences for changing marine ecosystems.

## MATERIALS AND METHODS

### Maintenance of *E. huxleyi* culture

*E. huxleyi* strain CCMP 2090 was cultivated in F/2-Si medium (*63*, *64*) prepared as follows: Seawater from an active marine aquarium (Aqua Vala, Brno, Czech Republic) was aged in the dark at 15°C for 2 weeks, passed through a 0.22-μm filter (Techno Plastic Products, Trasadingen, Switzerland), and further processed by tangential flow filtration [30-kDa molecular weight cut-off (MWCO), polyethersulfone membrane; Pellicon XL 50; Millipore, Merck, Darmstadt, Germany], autoclaved, and enriched with micronutrients (table S3). *E. huxleyi* cultures were inoculated to a final cell density of 2 × 10^5^ cells ml^−1^ in 600-ml tissue culture flasks (Jet BioFil, Guangzhou, China) and incubated in temperature- and illumination-controlled chambers (Photon Systems Instruments, Drásov, Czech Republic) at 15°C; light intensity of 50 μmol photons m^−2^ s^−1^ from light-emitting diodes with spectral ratios of white (33.3%), red (33.3%), and far-red (33.3%); and a 16-hour light/8-hour dark cycling regime with constant shaking (100 rpm, orbital shaker; N-Biotek, Bucheon, Republic of Korea). The cell density was measured using an automated cell counter (TC-20, Bio-Rad Laboratories, Hercules, California, USA) or by manual counting using a Bürker chamber (depth, 0.1 mm; Thermo Fisher Scientific, Waltham, Massachusetts, USA).

### EhV-201 production and infectivity assays

EhV-201 (*16*) (GenBank, accession code JF974311) was propagated on *E. huxleyi* strain CCPM 2090. An exponentially growing algal culture of *E. huxleyi* CCMP 2090 at a cell density of 1 × 10^6^ cells ml^−1^ was infected with EhV-201 at an MOI of 0.01 and left until complete lysis (up to 1 week). Viral stock solutions were prepared from the lysed algal culture by filtration through 0.22-μm syringe filters (Corning, New York, USA). The number of infectious viral particles was determined using plaque assay: 18 ml of an exponentially growing algal culture at a cell density of 1 × 10^6^ cells ml^−1^ was mixed with 100 μl of 10× serial dilutions of virus inoculum, incubated for 30 min at room temperature, mixed with 3% w/v low melting point agarose (UltraPure, Invitrogen, Thermo Fisher Scientific) in F/2-Si medium to a final concentration of 0.3%, and poured into petri dishes (diameter, 100 mm; Merck, USA). The cultures on petri dishes were incubated in a translucent plastic box under the same conditions as the liquid algal cultures (*65*). Plaques, cleared round areas within the algae layer, were counted 7 days after infection.

### EhV-201 purification and preparation for cryo-EM single-particle analysis

Two liters of an exponentially growing *E. huxleyi* CCMP 2090 at a cell density of 1 × 10^6^ cells ml^−1^ were infected with EhV-201 at an MOI of 0.01 and left until complete lysis (up to 1 week). For all subsequent purification steps, the lysate was kept on ice or at 4°C. The lysate was sequentially filtered through a tangential flow filtration cassette with 0.45-μm pore size (polyvinylidene difluoride membrane; Pellicon XL 50, Millipore) and concentrated on a 1000-kDa MWCO tangential flow filtration cassette (regenerated cellulose membrane; Pellicon XL 50, Millipore). The concentrate was further concentrated using a centrifugal ultrafiltration unit (100-kDa MWCO, regenerated cellulose; Amicon, Merck) to 200 μl at 600*g*. The resulting concentrate was applied to a 10 to 50% v/v iodixanol step gradient with 10% increments (OptiPrep; Sigma-Aldrich, Merck) enriched with sea salts (Sigma-Aldrich) to a final concentration of 600 mM to maintain the salinity of F/2-Si medium. Gradients were centrifuged in an ultracentrifuge (Optima XPN-80, Beckman Coulter, Danaher Corporation, Washington, DC, USA) using an SW-41 Ti rotor (Beckman Coulter) at 50,000*g* and 10°C for 60 min. The 30 to 40% interface band was extracted using a needle and syringe (B. Braun, Melsungen, Germany) and dialyzed twice 1:1000 against aqueous sea salt solution (40 g liter^−1^; Sigma-Aldrich) in dialysis sleeves (15-kDa MWCO; Roth, Karlsruhe Germany). The dialyzed virus particles were concentrated in centrifugal ultrafiltration units to 200 μl and mixed with Turbo Nuclease (Abnova, Taipei, Taiwan) at a final concentration of 25 U ml^−1^ to remove contaminating DNA and thus improve quality of the sample for cryo-EM. Sodium azide (Sigma-Aldrich) was added to a final concentration of 100 μg ml^−1^ to prevent bacterial growth. The purified virus was applied onto glow-discharged electron microscopy grids covered with holey carbon (Quantifoil, SPT Labtech, Melbourn, UK), blotted, and plunge-frozen using a Vitrobot Mark IV (Thermo Fisher Scientific) (table S4).

### Cryo-EM data collection and single-particle analysis

Data for single-particle analysis were acquired using a Titan Krios G2 cryo-TEM equipped with a Falcon 3EC direct electron detector (Thermo Fisher Scientific, Waltham, Massachusetts, USA) operating at 300 kV and a magnification at specimen level corresponding to a pixel size of 2.27 Å (table S1) controlled with the software EPU 1.8 (Thermo Fisher Scientific). Motion correction of the original movies was done using MotionCor2 (*66*), CTF estimation of the aligned micrographs was performed using gCTF 1.06 (*67*), and particles were automatically picked using SPHIRE-crYOLO 1.7.5 (*68*) trained on a manually picked small dataset. The particle images were extracted from twofold binned micrographs with a 128-pixel (px) box size and subjected to two-dimensional (2D) reference-free classification with a 500-Å-diameter mask using Relion 3.1 (*69*). Class averages corresponding to either the virion vertices or the rounded surface areas, respectively, were selected for further analysis. To plot the cryo-EM density values in the virion surface layers, pixel intensities along lines perpendicular to the particles’ surface were measured in the 2D class averages using ImageJ 1.44 (*70*). Angular virion vertices were selected by 3D classification using an initial model generated by the stochastic gradient descent method as implemented in Relion 3.1 (*69*). Subsequent refinement was performed using local searches with 1.8° sampling rate around the refined coordinates from 3D classification and applying a mask covering the capsid and outer membrane surface layers (fig. S18). The 1.8° rotational sampling is the recommended value for localized angular searches for low-symmetry object reconstructions in Relion. In this case, it corresponded to an 8-Å resolution limit, which is adequate considering the resolution of the resulting average.

### EhV-201 production, purification, and preparation for cryo-ET

A viral lysate was prepared in the same manner as the virions used for single-particle reconstruction, except for the addition of ampicilin and streptomycin (P-Lab, Praha, Czech Republic) (final concentrations of 100 μg ml^−1^) during algae culture cultivation and EhV-201 infection. Because the growth of the contaminating bacteria was reduced by the antibiotics, the OptiPrep step gradient and subsequent purification steps were omitted. Gold fiducials (6 nm; BSA tracer; Aurion, Wageningen, The Netherlands) were buffer exchanged into sea salts of 40 g liter^−1^ (Sigma-Aldrich) using centrifugal ultrafiltration unit (100-kDa MWCO; regenerated cellulose, Amicon) at 14,000*g* to the original volume. The centrifugal ultrafiltration concentrate of EhV-201 particles was mixed in a 3:1 ratio with the gold fiducials in sea salts. The final sample was applied onto electron microscopy grids covered with a holey carbon layer (Quantifoil), blotted, and plunge-frozen using a Vitrobot Mark IV (Thermo Fisher Scientific) (table S4).

### Cryo-ET tilt series data collection, reconstruction, and sub-tomogram averaging

Tilt series were collected using a Titan Krios G2 cryo-TEM (Thermo Fisher Scientific) equipped with a K3 direct electron detector and energy filter (Gatan, Ametek, Berwyn, Pennsylvania, USA) operating at 300 kV at a magnification corresponding to a pixel size of 2.08 Å at specimen level (table S1). Data were acquired using SerialEM 4.0 (*71*) and the protocol by Turonova *et al.* (*72*), with the following modifications: dose symmetric scheme starting from 0° with a 3° increment up to the maximum tilts of ± 48° (table S1). Original movies were motion-corrected using Warp 1.0.9 (*73*), and tilt series were aligned using the IMOD 4.10.45 package (*74*). Viral vertices were picked using template matching in emClarity 1.5.3 (*75*) with the virion vertex from single-particle reconstruction as a reference structure. Sub-tomograms were extracted using Warp (*73*) from twofold binned images with a box size of 128 px and imported into Relion 4.0 (*76*). Extensive 3D classification was performed using the virion vertex from the single-particle reconstruction as the initial model, applying a 500-Å-wide circular mask and an additional mask covering the capsid and outer membrane or only the capsid, respectively. Final 3D refinement was performed with local searches with 1.8° rotational sampling rate around the refined orientations from the 3D classification step and applying the same mask (fig. S19). To achieve overlap between the density maps of neighboring vertices, the sub-volumes were re-extracted using threefold binning with a box size of 208 px. The larger vertices were reconstructed using local searches around coordinates from 500-Å 3D refinement with 1.8° rotational sampling and applying a circular 1200-Å-diameter mask and mask covering the capsid and outer membrane.

### Determination of EhV-201 virion *T*-number and generation of composite virion map

The *T*-number of the EhV-201 capsid was calculated by placing three sub-tomogram reconstructions of the vertices, calculated using a 1200-Å-diameter mask, into the tomographic volume at the positions and orientations determined from 3D refinement, using in-house developed scripts. The *h* and *k* values were counted as the number of steps along capsomers required to connect the pentons of two neighboring, partially overlapping vertex volumes.

To generate the composite map of the EhV-201 virion with idealized icosahedral symmetry, the density map of the vertex was rotated and translated to maximize the cross-correlation among its overlapping copies related by a threefold symmetry axis from the set of icosahedral symmetry axes in standard orientation, using an in-house developed script. The idealized composite map of the EhV-201 virion was prepared by expanding the aligned vertex density map according to the icosahedral symmetry.

### Sample preparation for cryo-FIBM

An exponentially growing culture of *E. huxleyi* CCMP 2090 at a cell density of 1 × 10^6^ cells ml^−1^ was infected with EhV-201 at an MOI of 10, and the infection was allowed to progress for 48 hours. The infected cells were pelleted at 2000*g* for 10 min and resuspended in fresh F/2-Si medium at a final cell density of 1 × 10^7^ cells ml^−1^. Cells were applied onto electron microscopy grids covered with a holey gold layer (UltrAuFoil, Quantifoil, Jena Bioscience). The grids were flash-frozen using a Vitrobot Mark IV (Thermo Fisher Scientific) with backside blotting, for which the front-size blotting paper was replaced with a hydrophobic filter paper prepared in-house by soaking it in candle wax (table S4).

### Cryo-lamellae preparation, tomographic data acquisition, and analysis

The cryo-lamellae were produced by cryo-FIBM using a Versa-3D dual beam microscope equipped with a gallium ion source (Thermo Fisher Scientific) and cryo–transfer chamber (Quorum Technologies Ltd., Lewes, UK). The sample was sputtered with conductive inorganic platinum (30 s at 10 mA) to produce 5- to 8-nm-thick layer and coated with ~200-nm-thick protective organic platinum layer (methylcyclopentadienyl platinum precursor, 15 s, cryo-deposited by the gas injection system heated to 28°C). Lamellae ranging in width from 5 to 8 μm were produced from clusters of vitrified cells. The rough lamella shape was achieved by parallel milling patterns with Ga^2+^ ions at 30 kV and 0.5 nA with a stage tilt of 15° (ion beam at 8° angle relative to the grid). The subsequent thinning and polishing of lamellae to a final thickness of less than 300 nm was performed at 100 and 10 pA, respectively. Lamellae were transferred to a Titan Krios G2 cryo-TEM (Thermo Fisher Scientific) equipped with a K2 direct electron detector with an energy filter (Gatan). Tilt series were collected using SerialEM 4.0 (*71*) and a dose-symmetric scheme starting from a pre-tilt of 8° with a 3° increment, covering relative tilt angles from −45° to +45° at a constant defocus of −25 μm. Magnification and the resulting pixel size of each data collection varied from 7.4 to 13 Å.

Tomograms of cryo-lamellae were reconstructed using IMOD 4.10.45. The individual tilts were aligned using patch tracking, and the final binned tomograms were reconstructed at pixel sizes of 26 to 30 Å (*74*). The diameters of the viral particles and assembly intermediates were measured by fitting a circle around the most distal points of the virion using ImageJ 1.44 (*70*).

### Block-face imaging of resin-embedded algae

An exponentially growing culture of *E. huxleyi* CCMP 2090 at a cell density of 1 × 10^6^ cells ml^−1^ was concentrated as described in the cryo-lamellae FIBM preparation section and resuspended in the F/2-Si medium at a cell density of 1 × 10^9^ cells ml^−1^. Cells were mixed with EhV-201 concentrate prepared in the same manner as that for sub-tomogram averaging (without the addition of sodium azide) to obtain an MOI of 10. The mixture was incubated for 30 min at room temperature. Cryo-samples were prepared using high-pressure freezing in 200-μm carriers (cavity volume 0.6 mm^3^) treated with 0.2% w/v lecithin in chloroform (Sigma-Aldrich) using an EM ICE High Pressure Freezer (Leica Microsystems GmbH, Wetzlar, Germany) at 2010 bar. Cryo-preserved samples were freeze-substituted with 1% w/v osmium tetraoxide (Sigma-Aldrich, catalog no. 45345) in acetone (Penta, Chrudim, Czech Republic) using an EM AFS2 device (Leica Microsystems) using protocol: −90°C for 16 hours; −90° to −85°C for 6 hours; −85° to −60°C for 6 hours; −60°C for 5 hours; −60° to −30°C for 6 hours; −30°C for 4 hours; and − 30° to +10°C for 6 hours. Sample was further post-stained with 1% uranyl acetate (Electron Microscopy Science, PA, USA); infiltrated with epoxy embedding medium (Sigma-Aldrich) at epoxy:acetone ratio of 30:70 for 2 hours, 50:50 for 2 hours, and 70:30 for 2 hours; and washed four times with a fresh Epoxy medium for 12 hours. Polymerization was allowed to progress at 65°C for 48 hours. Resin blocks were mounted on an aluminum scanning electron microscopy stub and sputter-coated with 5-nm platinum using a Quorum Q150T (Quorum Technologies Ltd.). The serial block milling and imaging were performed using a Helios Hydra 5 CX with Auto Slice & View v. 4.2 (Thermo Fisher Scientific). A platinum protective layer (methylcyclopentadienyl platinum precursor) was deposited by a gas injection system on top of the region of interest to a total thickness of 1 μm at 30 kV and 1 pA. Trenches were milled using a xenon plasma beam at 30 kV and 15 nA, resulting in a final block size of 45 μm by 45 μm by 50 μm. To reduce the curtaining artifact, oxygen plasma (30 kV and 30 pA) was used for serial milling. Slices of a final size of 45 μm by 50 μm by 20 nm (*x*, *y*, *z*) were imaged using an in column detector in immersion mode (2 kV and 200 pA, dwell time of 10 μs). The resulting images had the dimensions of 3072 × 2048 px (*x*, *y*) with a pixel size of 1.69 nm. The final data cube was aligned, stitched without further post-processing, and analyzed using ImageJ 1.44 (*70*).

### Fluorescence microscopy

Exponentially growing cultures of *E. huxleyi* CCMP 2090 at a cell density of 1 × 10^6^ cells ml^−1^ were concentrated as for cryo-FIBM sample preparation and resuspended in F/2-Si medium at a cell density of 1 × 10^7^ cells ml^−1^. Concentrated EhV-201 virions, prepared as for sub-tomogram averaging (without the addition of sodium azide), were stained overnight with 4′,6-diamidino-2-phenylindole (DAPI; Roche, Basel, Switzerland) at a final concentration of 5 μg ml^−1^. Excess stain was removed by dialysis at a 1:1000 ratio against aqueous sea salt solution (40 g liter^−1^; Sigma-Aldrich) using dialysis sleeves (15-kDa MWCO; Roth) at 4°C in the dark. Algae were mixed with EhV-201 at MOIs of 10 and 100, respectively. The mixtures were incubated at room temperature in the dark for 30 min and stained with *N*-(3-triethylammonium propyl)-4-[4-(dibutyl amino) styryl] pyridinium dibromide (FM 1-43FX, Invitrogen, catalog no. F35355) at a final concentration of 5.6 μg ml^−1^. After 5 min of incubation, the mixture was fixed using glutaraldehyde (Penta, Chrudim, Czech Republic) at a final concentration of 0.2% v/v and immediately transferred onto a 10-well cell culture microscopy slide (CellView; Greiner Group AG, Kremsmünster, Austria) pretreated with poly-lysine (Sigma-Aldrich) dissolved in distilled water at a concentration of 0.01% w/v. After a 30-min settling time, the supernatant was removed, and the wells were covered with mounting medium (ProLong glass antifade, Invitrogen).

Fluorescence images were recorded using an Elyra 7 super-resolution microscope operated in lattice structured illumination microscopy (SIM) mode and controlled using the ZEN black edition 3.0 system (ZEISS, Oberkochen, Germany). Volume data were acquired using an oil immersion objective (Plan-Apochromat 63×/1.4 Oil DIC) and detected with a pco.edge sCMOS camera using a frame size of 512 px (*x*, *y*) with nine-phase grating in leap mode using the following channel settings: DAPI: 405-nm laser 20% excitation, BP420-480 dichroic mirror and SBS BP 490-560 beam splitter, and exposure time of 50 ms; FM 1-43: 488-nm laser 1% excitation, 495-590 dichroic mirror and SBS BP 490-560 beam splitter, and exposure time of 30 ms. Acquired volume data were reconstructed with ZEN black edition 3.0 (ZEISS) using the 3D SIM^2^ method with drift correction measured using fluorescent beads.

The distance of the virus particle from the algal cell surface was determined by subtracting the radius of a circle fitted around the circumference of the (FM 1-43–stained) algal plasma membrane from the larger circle with the same origin crossing the viral DAPI-stained genome. The cutoff distance for attachment was set to 300 nm, i.e., 1.5 times the virus particle diameter.

### Reconstruction and segmentation of tomograms of infected *E. huxleyi* cells

Tilt series of cryo-lamellae were reconstructed using the package IMOD 4.10.45 (*74*). Selected tomograms of infected and noninfected control cells were segmented using artificial intelligence-assisted segmentation as implemented in DragonFly 2022.2.0 (Object Research System, Montréal, Québec, Canada). The network was trained on a small portion of manually segmented tomogram volume and applied to the entire tomogram, followed by extensive manual pruning. The positions of EhV-201 virions and full particles were determined by template matching using emClarity 1.5.3.11 (*75*) and composite maps of EhV-201 virions with and without the outer membrane, respectively, were placed back into the tomographic volume using in-house developed scripts. Images and movies of the segmented tomograms were generated using ChimeraX 1.5 (*77*).

### Size comparison of distinct EhV-201 assembly intermediates

Statistical analyses were performed using the R v4.2.2 in RStudio environment v2022.07.2 and the following libraries: emmeans_1.8.1-1, car_3.1-0, carData_3.0-5, lme4_1.1-30, and Matrix_1.5-1 (*78*–*80*). Data visualization was performed using ggplot2_3.3.6 (*81*). The maximum-outer diameters of virus particles were measured in tomographic reconstructions of infected *E. huxleyi* cells: genome packaging intermediate (*N* = 25), full particle (*N* = 25), and virion (*N* = 25) in triplicates and averaged. The diameter of each particle was measured at different *Z*-heights to determine the maximum diameter. Particle diameter was treated as a continuous response variable, and assembly stage was treated as a fixed factor. The Dataset_ID (three levels) of different sample preparations and data collections were treated as random factors because Cell_ID (nine levels), an identifier of individual cells in the cryo-lamellae, did not contain all the assembly stages equally distributed. The diameter of particles was compared by a linear mixed model with Dataset_ID as a random factor. The H0 of equal particle diameter was rejected by analysis of variance (ANOVA Type-II) at *P* < 0.0001 (df = 73, *F* = 229.6). Pairwise analysis of diameter between intermediate stages to determine the *P* value was done by multiple analysis of means.

### Effect of DAPI staining on EhV-201 infectivity

The titer of a viral lysate was determined from the concentration of plaque-forming units using plaque assay (*N* = 3). The concentration of plaque-forming units was treated as a continuous response variable and DAPI treatment as a predictor variable. The concentrations of plaque-forming units were compared using the Welch two-sample (two-tailed) *t* test, and the H0 of an equal number of plaques was not rejected at *P* = 0.195 (df = 4, *t* = 1.63).

### Bioinformatic analysis of EhV-201 penton protein

Annotation of putative penton protein of EhV-201 was done using the complete genome of EhV-86 by PSIBLAST BLAST+ (v2.12) (*82*), (*e*-value cutoff of 0.1) using multiple sequence alignment of NCVs penton proteins as a query and all proteins from EhV-86 genome as a search subject. To build the penton profile long and short versions of PBCV-1 penton protein, P1v1 (NP 048889) and P1 (NP 048665), were used separately as queries in three iterations of the PSIBLAST search against National Center for Biotechnology Information (NCBI) “nr” database. The resulting hits were aligned using the MAFFT (v7.490) fast FFT2 algorithm (*83*). Redundant sequences with higher than 85% sequence similarity were removed using the Jalview2 alignment viewer (*84*). Given multiple crossovers between PSIBLAST results of PBCV-1’s long and short pentons, the two datasets were merged and aligned using the MAFFT G-INS-i algorithm with a limit of 1000 iterations. The final alignment was trimmed using trimAl (v1.4) (*85*) by removing columns with more than 20% gaps (file S1 “penton_ginsi_tr02GT.fasta”) and used directly for PSIBLAST search against EhV-86 proteins yielding a single hit (YP_293955, locus EhV201, *e* value of 0.017). The same basic approach was used to search for paralogs of major capsid protein gene in the EhV-86 genome using the PBCV-1 known major capsid protein paralogs as a query resulting in a single hit (YP_293839, *e* value of 5.15 × 10^−83^), which was known to be the major capsid protein.

### Mass spectrometry

Proteins from EhV-201 particles from the 30 to 40% v/v interface of the iodixanol step gradient (OptiPrep; Sigma-Aldrich, Merck) were extracted using chloroform-methanol precipitation. The resulting dried protein pellet was resuspended in denaturing Läemmli buffer and boiled for 3 min. Proteins were separated on discontinuous 12% in-house prepared SDS polyacrylamide gel running at 150 V for 1 hour in tris-glycine-SDS running buffer. The gel was washed twice for 10 min in ultrapure water, fixed with 25% isopropanol and 10% acetic acid solution for 30 min, and stained with fresh colloidal G-250 overnight, and the background was removed by repeated washing in ultrapure water. To select the bands for mass spectrometry (MS) analysis, the gel was further silver-stained, and prominent protein bands were cut from the gel and used for MS analysis. Liquid chromatography–tandem MS (MS/MS) analyses of peptide mixtures coming from in-gel digestions with sequencing-grade trypsin (Promega) were done using RSLCnano system (Thermo Fisher Scientific) online connected to Impact II Ultra-High Resolution Qq-Time-Of-Flight mass spectrometer (Bruker, Bremen, Germany). Preprocessing of data was carried out in DataAnalysis software (4.2 SR1; Bruker). Exported MS/MS spectra were analyzed in Proteome Discoverer software (Thermo Fisher Scientific; v1.4) with in-house Mascot (Matrixscience, London, UK; v2.4) search engine utilization. Mascot software (Matrix Science, London, UK) was used for sequence searches in exported MS/MS spectra against the NCBI database of *E. huxleyi* virus 86 (taxonomy ID: 181082) and *E. huxleyi* (taxonomy ID: 280463) and the common Repository of Adventitious Proteins contaminant database. The mass tolerance of peptides and MS/MS fragments for MS/MS ion searches were 50 parts per million and 0.5 Da, respectively. The oxidation of methionine and propionyl-amidation of cysteine as optional modifications and one enzyme miss-cleavage were set for all searches. Peptides with a statistically significant peptide score (*P* < 0.05) were considered.
