# Supplementary material for: Structure and replication cycle of a virus infecting climate-modulating alga Emiliania huxleyi
Source: Sci Adv. 2024 Apr 10;10(15):eadk1954. doi: 10.1126/sciadv.adk1954 (PMC11006232; doi:10.1126/sciadv.adk1954)
Supplement: Supplementary file 1 — Figs. S1 to S19 Tables S1 to S4 Legends for movies S1 to S3 Legend for file S1 [file sciadv.adk1954_sm.pdf]

Science Advances
AAAS

# Supplementary Materials for

## Structure and replication cycle of a virus infecting climate-modulating alga *Emiliania huxleyi*

Miroslav Homola *et al.*

Corresponding author: Pavel Plevka, pavel.plevka@ceitec.muni.cz

*Sci. Adv.* **10**, eadk1954 (2024)
DOI: 10.1126/sciadv.adk1954

**The PDF file includes:**

Figs. S1 to S19
Tables S1 to S4
Legends for movies S1 to S3
Legend for file S1

**Other Supplementary Material for this manuscript includes the following:**

Movies S1 to S3
File S1

**Supplementary figures:**

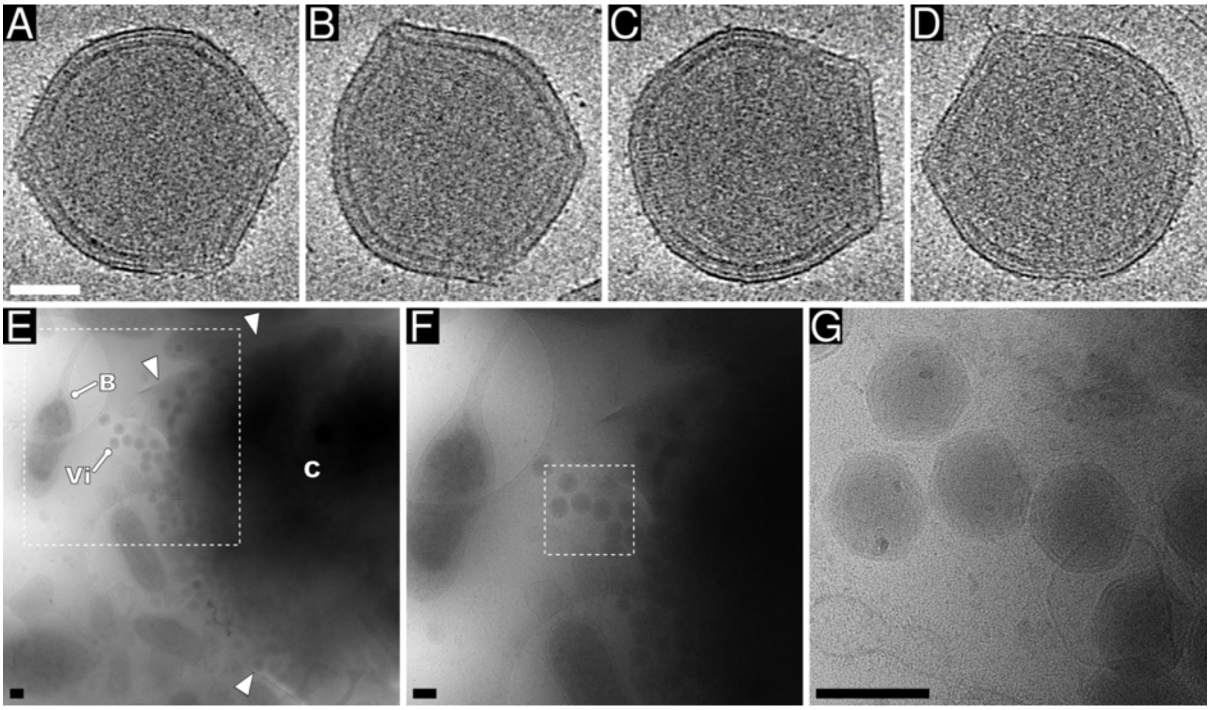


**Fig. S1. EhV-201 virions are pleomorphic. (A-D)** Projection images of 8-nm-thick sections from cryo-tomograms of EhV-201 virions, which show regions with sharp edges and angular vertices, but also rounded parts. The particles differ from each other. Scale bar 50 nm. **(E-G)** Cryo-electron micrographs of EhV-201-infected *E. huxleyi* cell that lysed during the vitrification of the sample for cryo-EM. Scale bar 200 nm. **(E)** A lysed *E. huxleyi* cell (C) with white arrowheads indicating the edges of the ruptured plasma membrane, B bacteria that grow in co-culture with *E. huxleyi*, Vi EhV-201 virions. The dashed square indicates the position of the region shown at a higher magnification in panel (F). **(F)** Intermediate magnification of virions released from ruptured *E. huxleyi* cell. The dashed square indicates the position of the region shown at higher magnification in panel (G). **(G)** EhV-201 virions are deformed and differ structurally from each other immediately after release from a lysed cell.

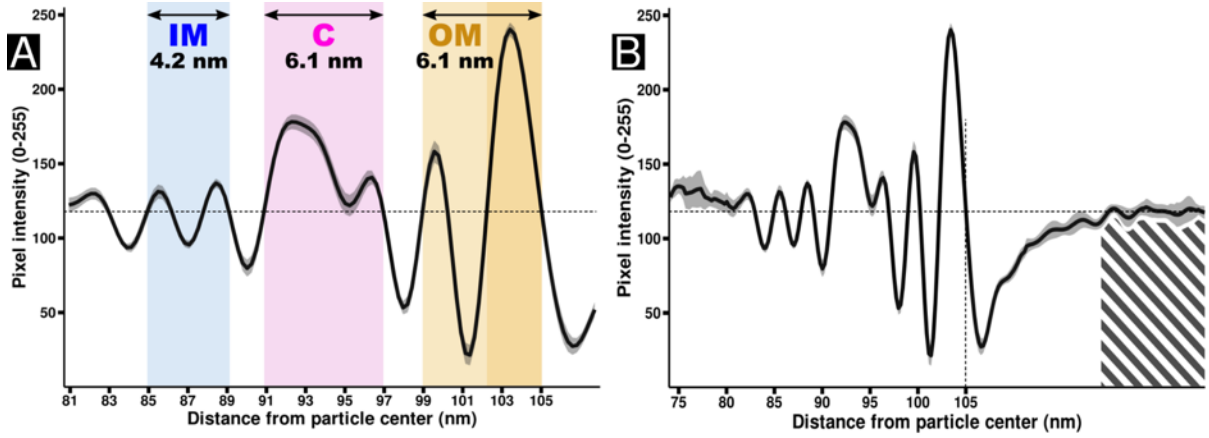


**Fig. S2. Surface layers of EhV-201 virion. (A)** Plot of average pixel intensities measured along lines perpendicular to particle surface in reference-free two-dimensional class average of oblique segments of EhV-201 particle surface. Layers representing the inner membrane, capsid, and outer membrane are indicated by colored backgrounds (IM inner membrane is shown in blue, C capsid in magenta, and OM outer membrane in orange). Numbers indicate layer thickness. The outer leaflet of the outer membrane, indicated by dark orange, has a stronger density than the inner leaflet. The coloring scheme corresponds to that in Fig. 1B, C. The 95% confidence interval is indicated by grey shading. The average background intensity level is indicated by a horizontal dashed line. **(B)** Extended plot including region used for determination of average background value calculated from hatched area. N = 18.

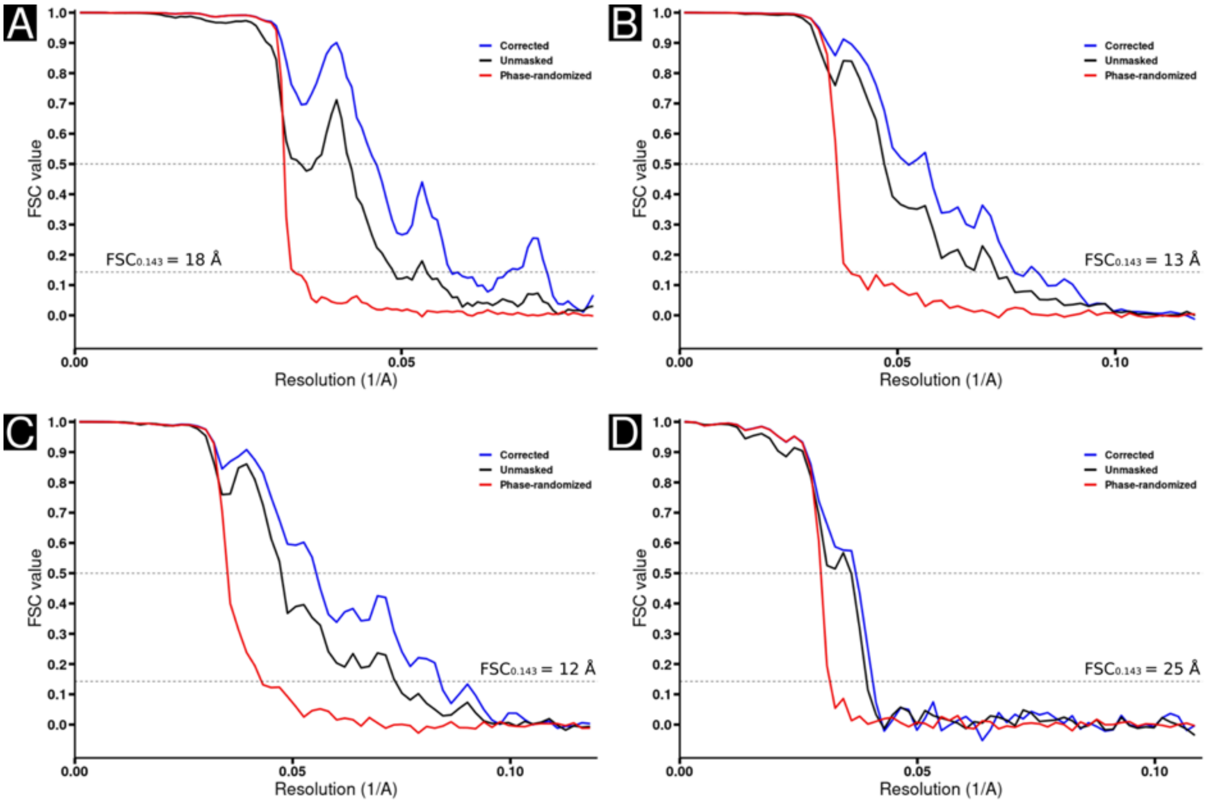


**Fig. S3. Plots of Fourier shell correlation (FSC) of reconstructions of independent halves of cryo-ET and cryo-EM datasets of EhV-201 virion vertices. (A,B)** Sub-tomogram reconstructions of EhV-201 virion vertex with masks limiting the size of the reconstruction to 120 nm (A) and 50 nm (B). **(C)** Sub-tomogram reconstructions of EhV-201 virion vertex with mask limiting the size of the reconstruction to 50 nm and removing the outer and inner membrane. **(D)** Single-particle reconstruction of EhV-201 virion vertex with masks limiting the size of the reconstruction to 50 nm. Dashed lines indicate FSC values 0.5 and 0.143.

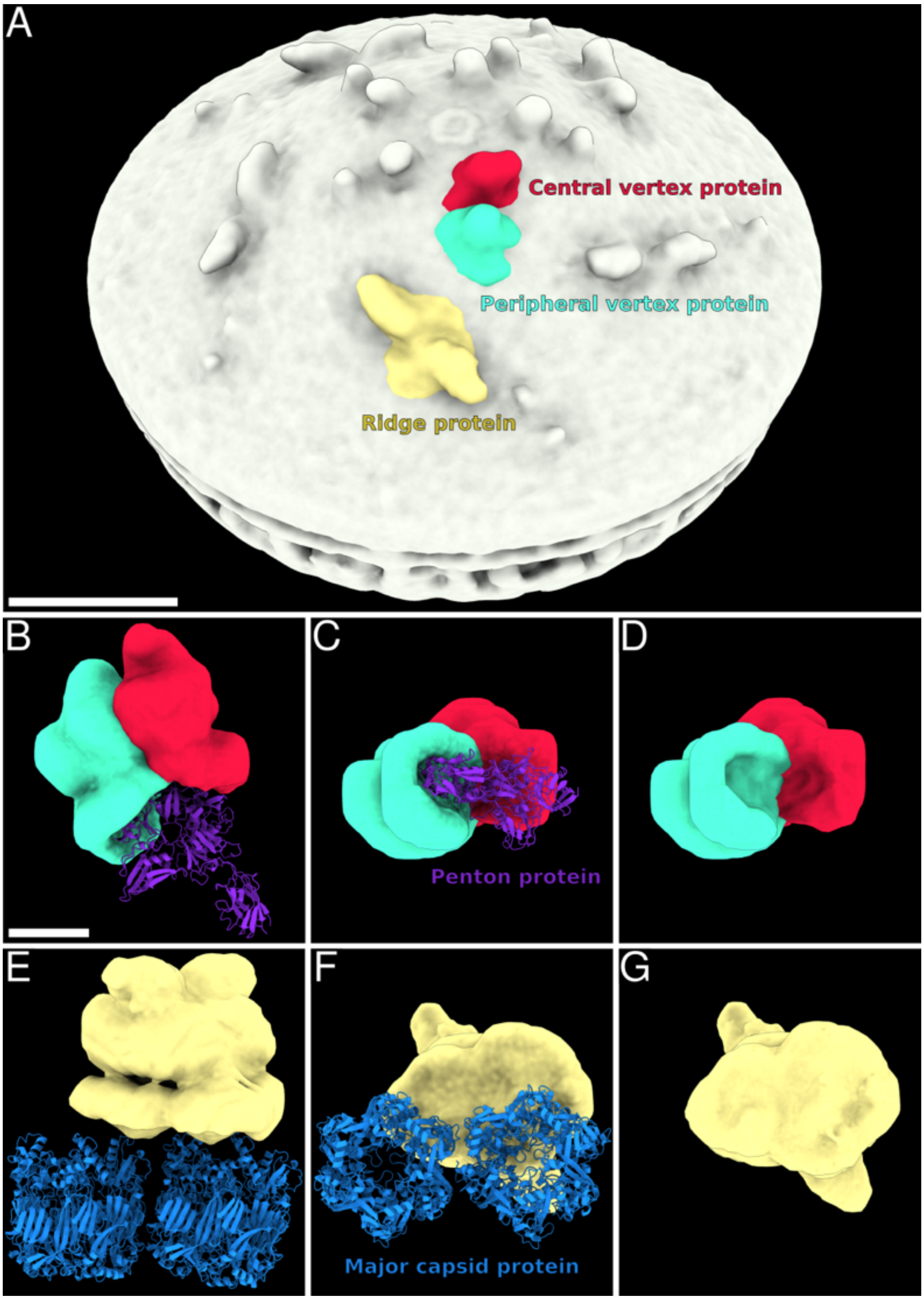


**Fig. S4. Outer membrane of EhV-201 is decorated with transmembrane proteins. (A)** Surface representation of sub-tomogram of EhV-201 vertex reconstructed using a mask with the diameter of 50 nm showing central vertex proteins (red), peripheral vertex proteins (light blue), and dimers of ridge proteins (yellow). Scale bar 10 nm. **(B-G)** Surface representations of transmembrane proteins in side (B,E) and bottom views (C,D,F,G). Central and peripheral vertex proteins (B-D) with underlying monomer of the penton protein in cartoon representation (purple) (B,C) and without it (D) – the cavity, where the penton protein binds is visible. Ridge protein dimer (E-G) with underlying major capsid protein capsomers in cartoon representation (blue) (E,F) and without the major capsid proteins (G). Scale bar (B-G) 5 nm.

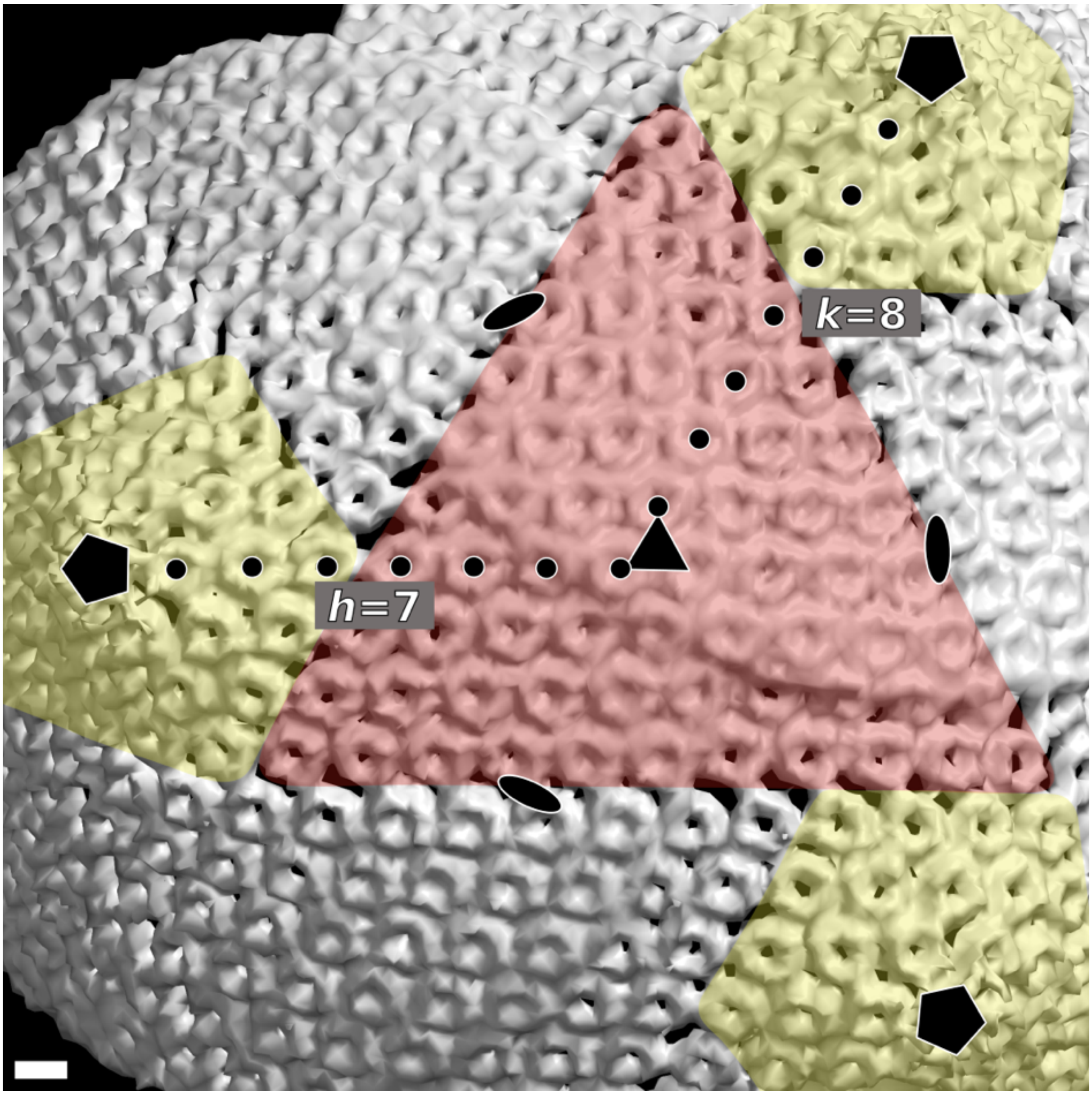


**Fig. S5. EhV-201 capsid is organized with *T* = 169 quasi-symmetry.** Surface representation of three cryo-ET reconstructions of angular vertices (mask diameter 120 nm) placed back into tomogram of EhV-201 virion based on coordinates obtained by three-dimensional refinement. Positions of fivefold symmetry axes are indicated by red pentagons, *h* and *k* directions are indicated by red dots. Selected penta-symmetrons are highlighted in yellow and tri-symmetron in red. Positions of selected fivefold symmetry axes are indicated by black pentagons, threefold axis by a triangle, and twofold axes by ovals. Scale bar 5 nm.

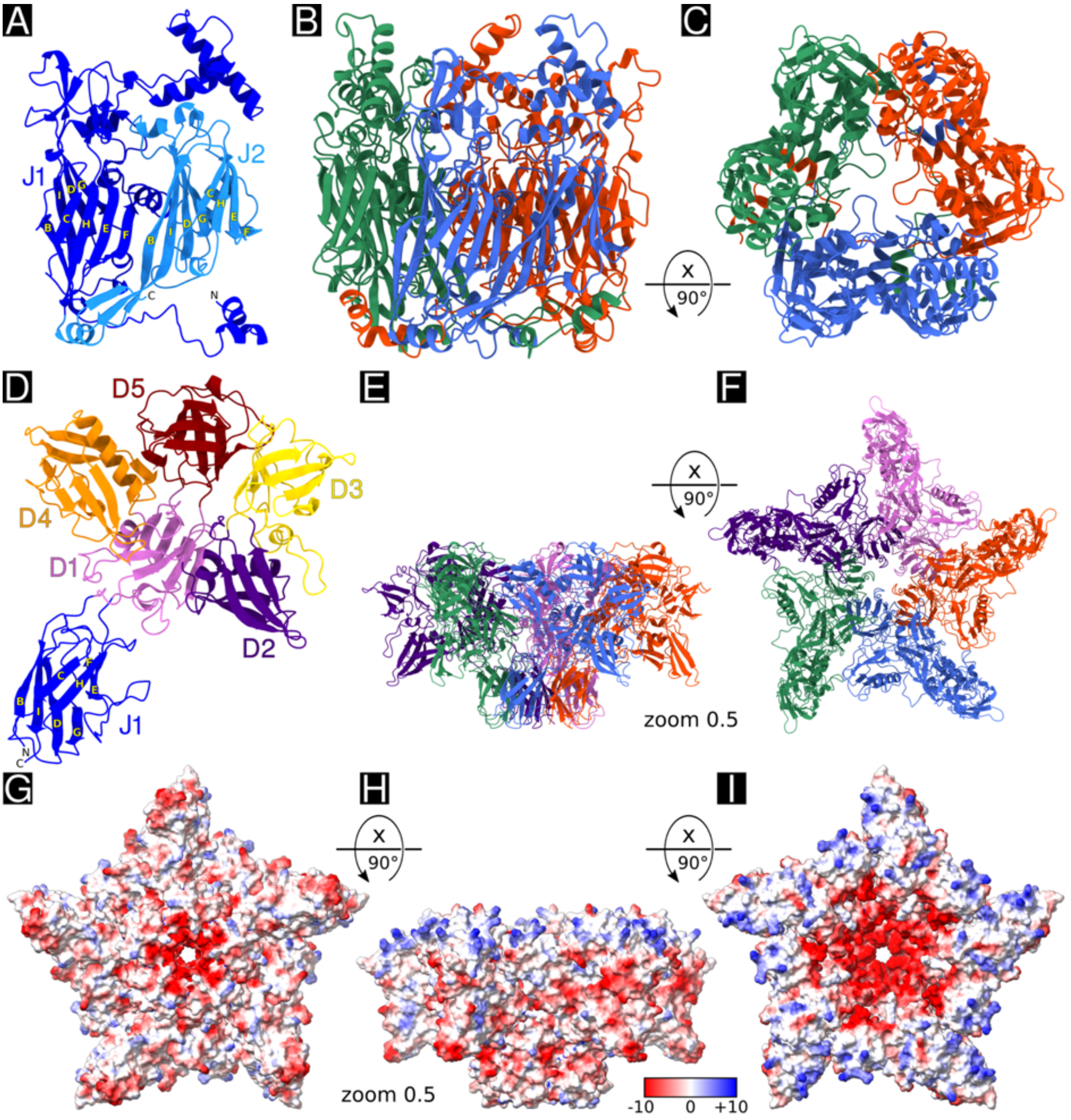


**Fig. S6. Structure of EhV-201 major capsid protein and penton protein. (A)** Cartoon representation of AlphaFold2 (*36*) predicted structure of a monomer of EhV-201 major capsid protein. The domains J1 and J2 are colored in dark and light blue, respectively. Each domain contains two four-stranded β-sheets with the β-strands conventionally named BIDG and CHEF. Domain J1 contains an insertion between β-strands D and E, DE loop, (grey) which contains amphipathic helices α3 (orange) and α4 (magenta). **(B,C)** Side (B) and top (C) view of capsomer formed by three monomers of the major capsid proteins shown in red, green, and blue. **(D-F)** Cartoon representation of EhV-201 penton protein. (D) A monomer of penton protein with single jelly roll fold domain J1 is shown in blue. The domain is formed by two four-stranded β-sheets with the β-strands named BIDG and CHEF. An insertion in the domain J1 forms domains D1-5, which are distinguished by colors (Table S2). (E, F) Pentamer of penton proteins, each of which is shown in distinct color. **(G-I)** Electrostatic surface potential plot of the penton protein pentamer. The surface of the penton exposed to the outside of the capsid is positively

charged. Negatively charged areas are colored in red, and positively charged areas in blue. Color key in kcal/mol at 298 K. Panels (E-I) are scaled down to 50 % to fit the figure dimensions. The central domain D1 connects the jellyroll and the four other globular insertion domains. The domains D1, D2, and D4 share a mixed $\alpha/\beta$-barrel fold of seven anti-parallel $\beta$-strands flanking an $\alpha$-helix (Table S2). Structural similarity search using DALI (*87*) determined that it is a common structural module in many viral penton insertion domains (*88*). The domains D3 and D5 share similarity with insertion modules of other virus proteins that have structural functions (Table S2) (*89, 90*).

A

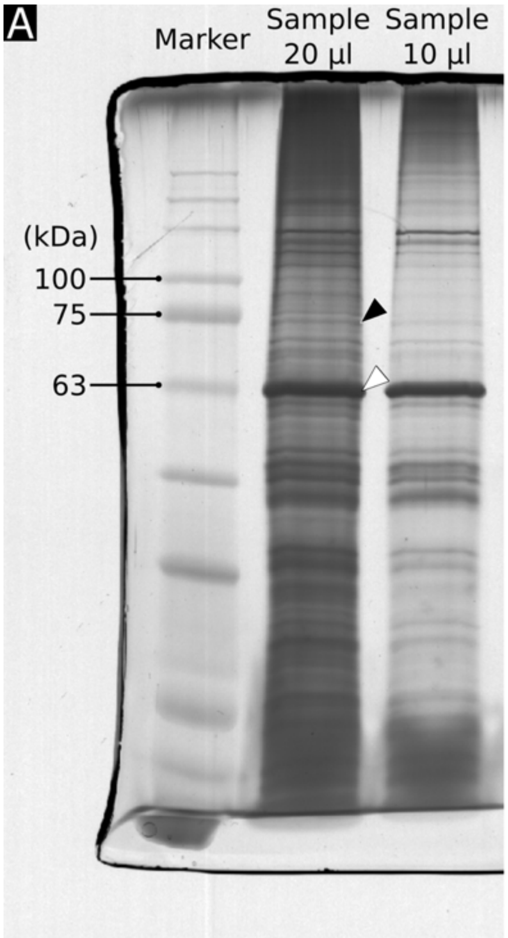


B

| Proteomic analysis of penton protein | |
|---|---|
| **Accession No.** | YP_293955.1 |
| **Protein description** | Hypothetical protein EhV201 [Emiliania huxleyi virus 86] |
| **MW [kDa]** | 72,7 |
| **Spectral Abundance Factor (SAF)** | 0,06 |
| **Normalized SAF (NSAF) (%)** | 9,76% |
| **Sequence coverage (%)** | 27,90% |
| **Sum of unique peptides** | 16 |
| **Sequence of detected unique peptides** | |
| ESSIIFR | |
| FNNALQQFK | |
| FTLNEGSSHR | |
| GDVVTLTTVMPAPDGATGTK | |
| GDVVTLTTVMPAPDGATGTKR | |
| GVSGYTWNVTLPSGNYGTPEK | |
| GYSGGTLR | |
| LGFSNGVSTGQHGVVVSQCAVNLEPR | |
| NVISSYK | |
| SIDFPYSQR | |
| SLITQVPSDR | |
| SSGPVPQGMVR | |
| SVSVLLTDK | |
| SVSVLLTDKESGDR | |
| TIESSWNK | |
| TVDVSFIEGSMTSASTEMYNQNGK | |

**Fig. S7. Mass spectrometry identification of the penton protein. (A)** A silver-stained SDS-PAGE gel of EhV-201 virion proteins. The band indicated by a white arrowhead contained major capsid protein. The band indicated by a black arrowhead contained the penton protein. **(B)** List of peptides from the penton protein that were identified using mass spectrometry analysis in the band indicated by the white arrowhead in panel A.

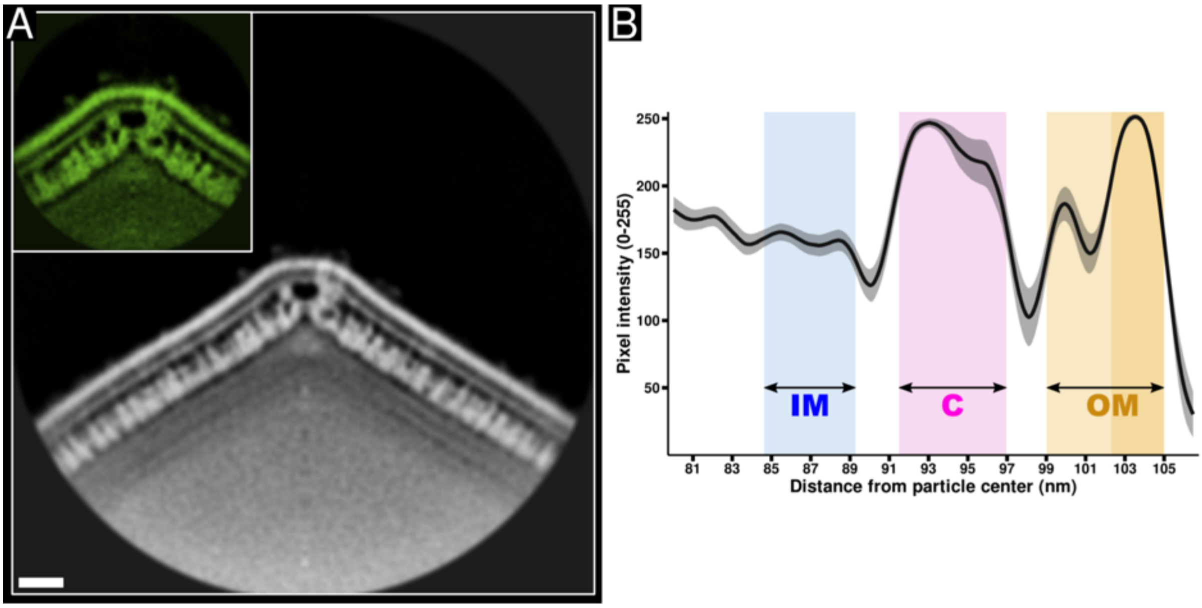


**Fig. S8. Inner membrane is less resolved than outer one in EhV-201 virion vertex sub-tomogram reconstruction. (A)** Central sections of sub-tomogram reconstructions of vertices from EhV-201 virion with reconstruction diameter set to 120 nm (grey) and 50 nm (inset in green). No feature-based masks were applied in the reconstruction process. Scale bar 10 nm. **(B)** Plot of average voxel intensities measured along lines perpendicular to EhV-201 virion surface. Layers representing the inner membrane, capsid, and outer membrane are marked by colored backgrounds (IM inner membrane in blue, C capsid in magenta, and OM outer membrane in orange). The coloring scheme corresponds to that in Fig. 1B,C. The 95% confidence interval (N = 14) is indicated by grey shading.

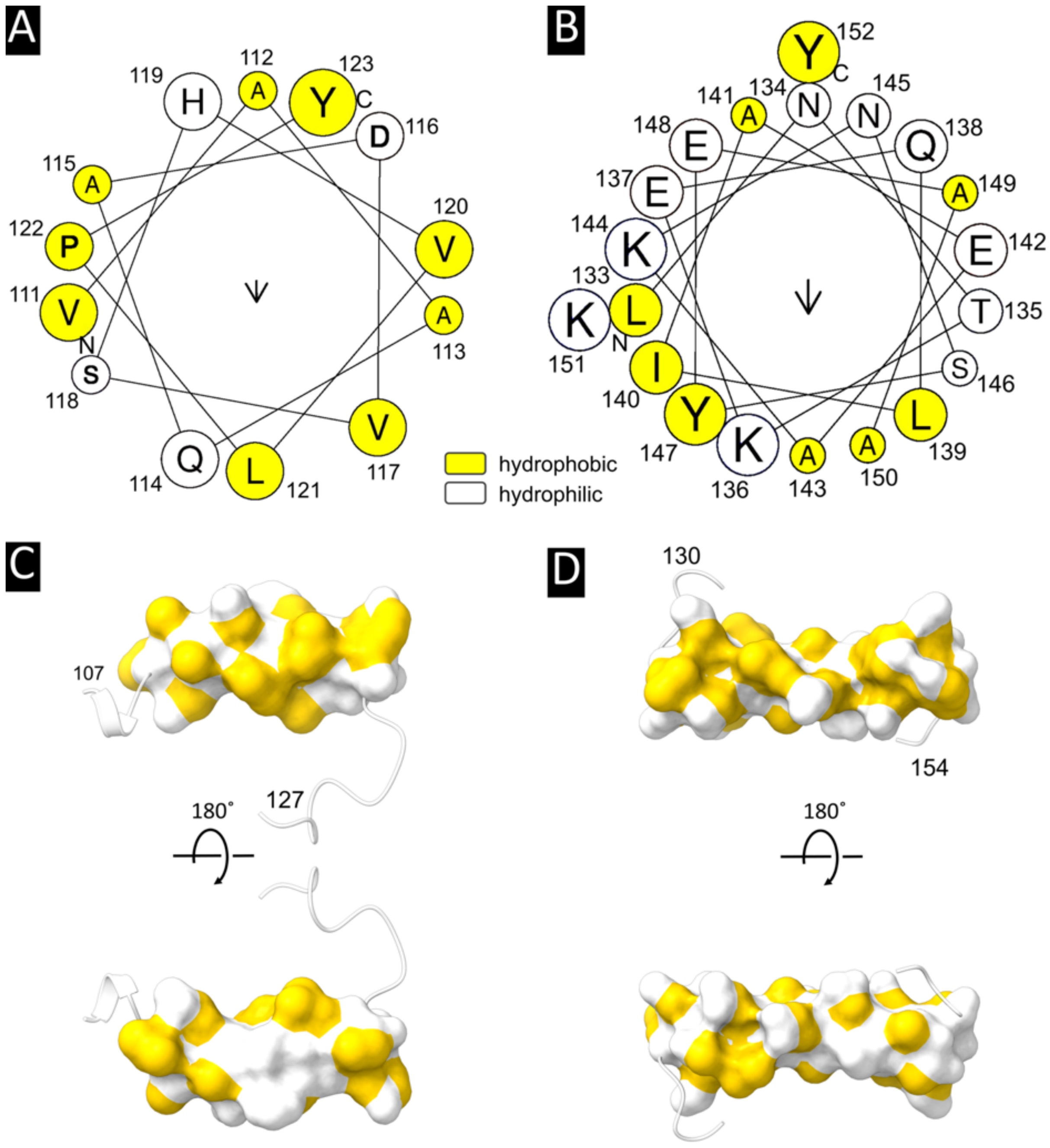


**Fig. S9. DE loop of J1 domain of EhV-201 major capsid protein contains amphipathic helices α3 and α4. (A)** Helical wheel representation of helix α3 (residues 111-123) from J1 domain of EhV-201 major capsid protein, prepared using HeliQuest server (*91*), indicating its amphipathic properties. Amino acids with hydrophobic side chains are shown in yellow and hydrophilic amino acids in white. **(B)** HeliQuest plot of helix α4 (residues 133-152). The arrows in panels (B) and (C) indicate the magnitude and direction of the hydrophobic moment. **(CD)** Surface representation of amphipathic helices α3 (C) and α4 (D) colored by hydrophobicity showing clustering of hydrophobic residues (yellow) on one face (top panel) whereas polar residues prevail on the opposite side (bottom panel).

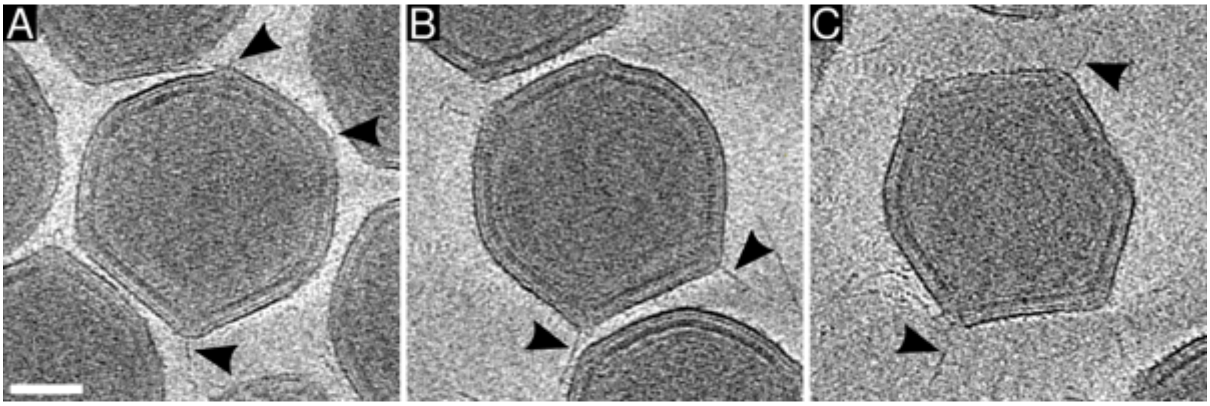


**Fig. S10. Some vertices of EhV-201 virions are decorated with flexible fibers. (A-C)** Projection images of 16-nm-thick sections of cryo-tomograms of EhV-201 virions. Fibers attached to some of the virion vertices are indicated by black arrowheads. Scale bar 50 nm.

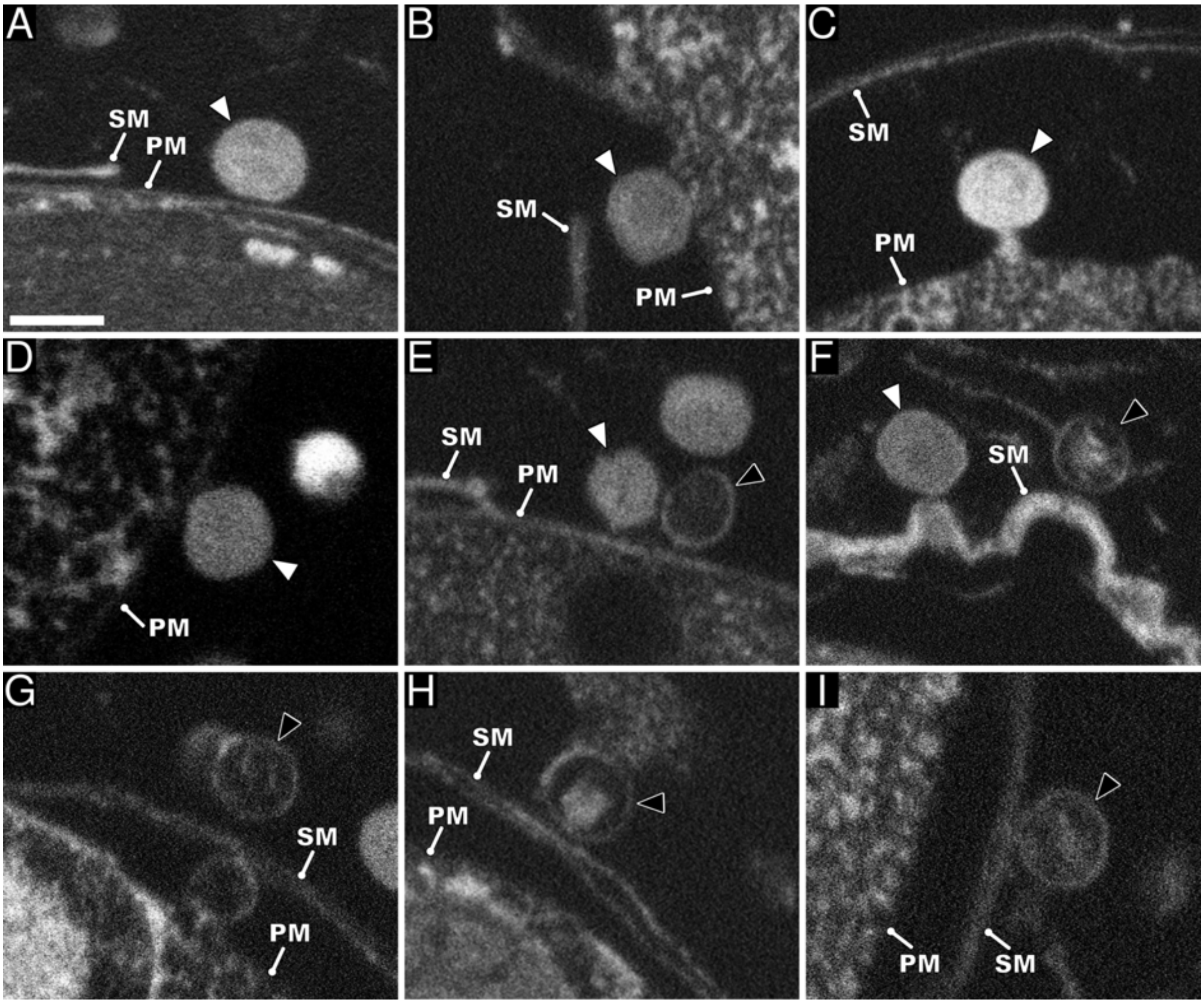


**Fig. S11. Productive and abortive genome delivery of EhV-201.** Scanning electron micrographs of a high-pressure vitrified and resin-embedded sample of *E. huxleyi* cells infected by EhV-201 at MOI = 10, 30 min post-infection. **(A-E)** Productive infection pathway. Genome-containing (white arrowhead) (A-E) and empty (black arrowhead) (E) EhV-201 particles attached to PM plasma membrane. **(F-I)** Abortive infection. Genome-containing (F) and empty (F-I) EhV-201 particles attached to SM surface membrane of *E. huxleyi* cells Please note that the cell envelope, which covers most *E. huxleyi* cells when imaged using cryo-electron microscopy (Fig. 3), is not resolved in the resin-embedded samples, probably because it was dissolved during the sample preparation procedure or not stained by osmium tetroxide and uranyl acetate used for sample contrasting. Scale bar 200 nm.

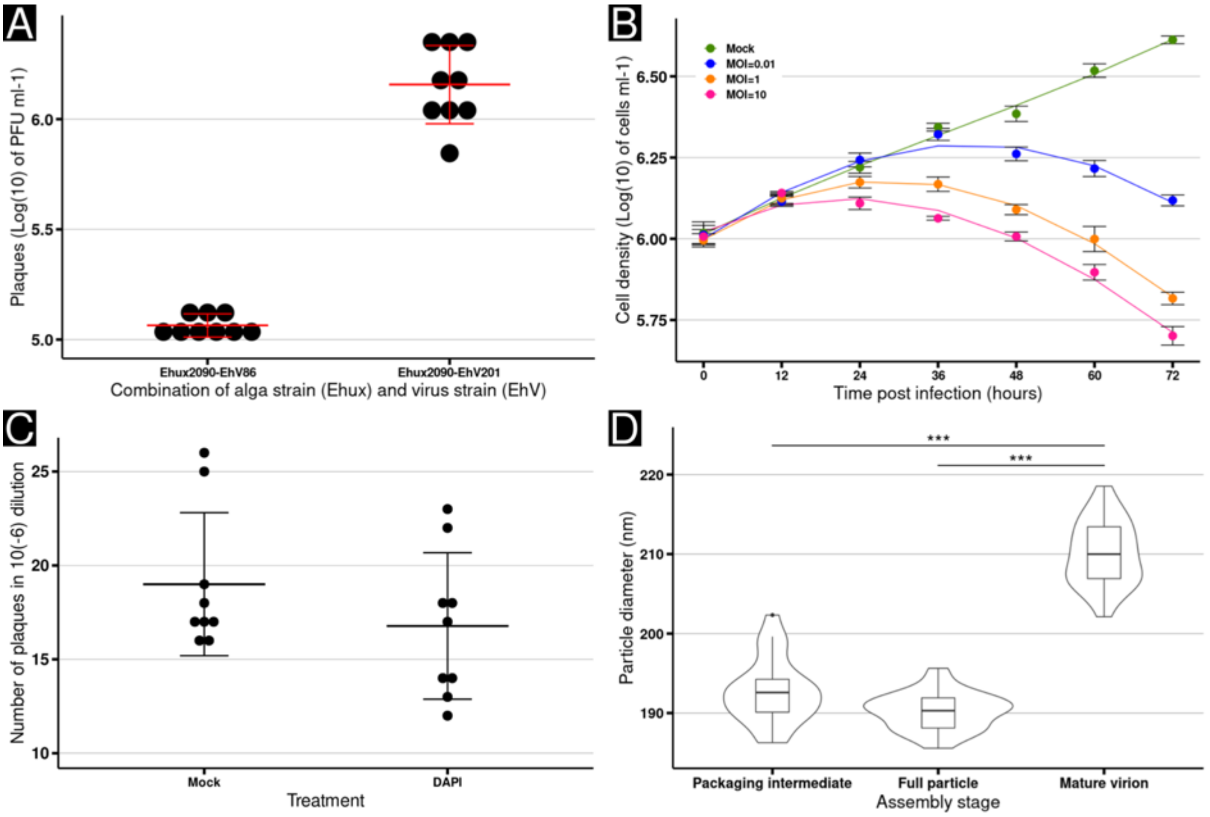


**Fig. S12. Properties of EhV-201. (A)** The efficiency of EhV-201 propagation on *E. huxleyi* strain CCMP 2090. Dot plot showing the number of plaque forming units per milliliter obtained for EhV-86 and EhV-201 propagated on *E. huxleyi* CCMP 2090. Mean and standard deviation (error bars) are indicated. N = 3. **(B)** Lysis of *E. huxleyi* culture by EhV-201 at various MOI. Growth curves of *E. huxleyi* CCMP 2090 infected by EhV-201 at MOI 0 (mock), 0.01, 1, and 10. Curves represent the 3rd-order polynomial fit to the data. Error bars correspond to the standard deviation (N = 3). **(C)** EhV-201 infectivity is not affected by DAPI fluorescence staining. Dot plot of the number of plaque-forming units in 100 µl of a viral lysate with and without DAPI treatment. The mean and standard deviation (error bars) are indicated. The seawater medium-treated group was used as a control (Mock). **(D)** Size distribution of EhV-201 assembly intermediates. The maximum outer diameters of genome packaging intermediates, full capsids, and virions were measured from cryo-tomograms of infected cells. Violin plots showing both kernel density and box plot: central black line - median; box - interquartile range; whiskers - 1st and 4th data quartile without outliers; the outlier greater than 1.5 times the interquartile range is depicted by a black dot. N = 25.

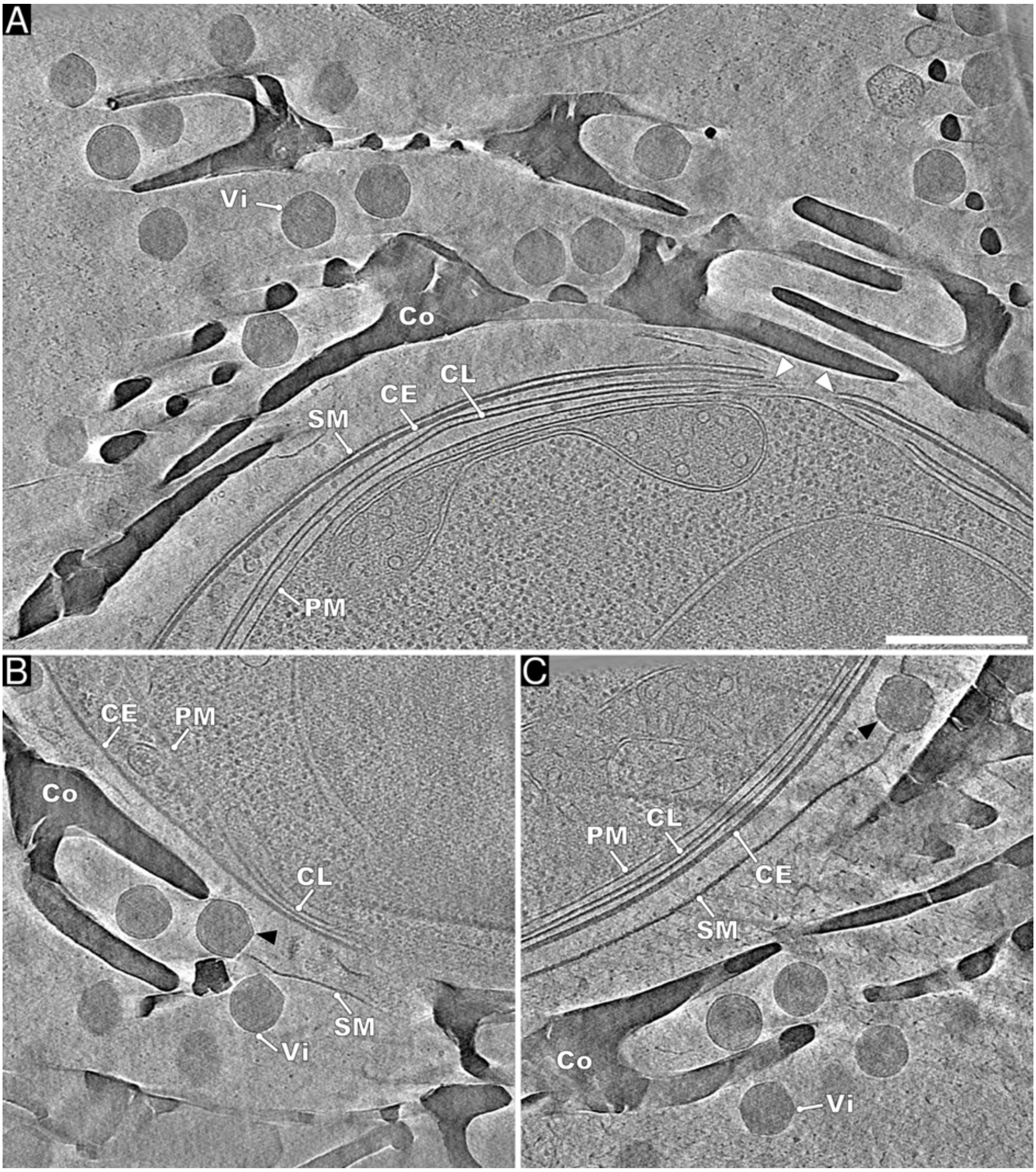


**Fig. S13. Surface layers of *E. huxleyi* cells. (A-C)** Projection images of 30-nm-thick tomogram sections of a cell from the non-calcifying *E. huxleyi* strain CCMP 2090, which spontaneously resumed coccoliths production. The cell is surrounded by a large number of EhV-201 virions (Vi) as it was infected at MOI = 100 and imaged at 30 min post-infection. Co coccolith, SM surface membrane, CE cell envelope, CL cytoplasmic leaflet, and PM plasma membrane. The opening in the surface membrane, cell envelope, and cytoplasmic leaflets is indicated by white arrowheads. (B,C) Virions of EhV-201 can diffuse beneath the *E. huxleyi* coccoliths shell as indicated by black arrowheads. The particles have diameter 209 nm SD 4.3 nm, indicating that they are virions containing outer membrane (N = 19). Scale bar 500 nm.

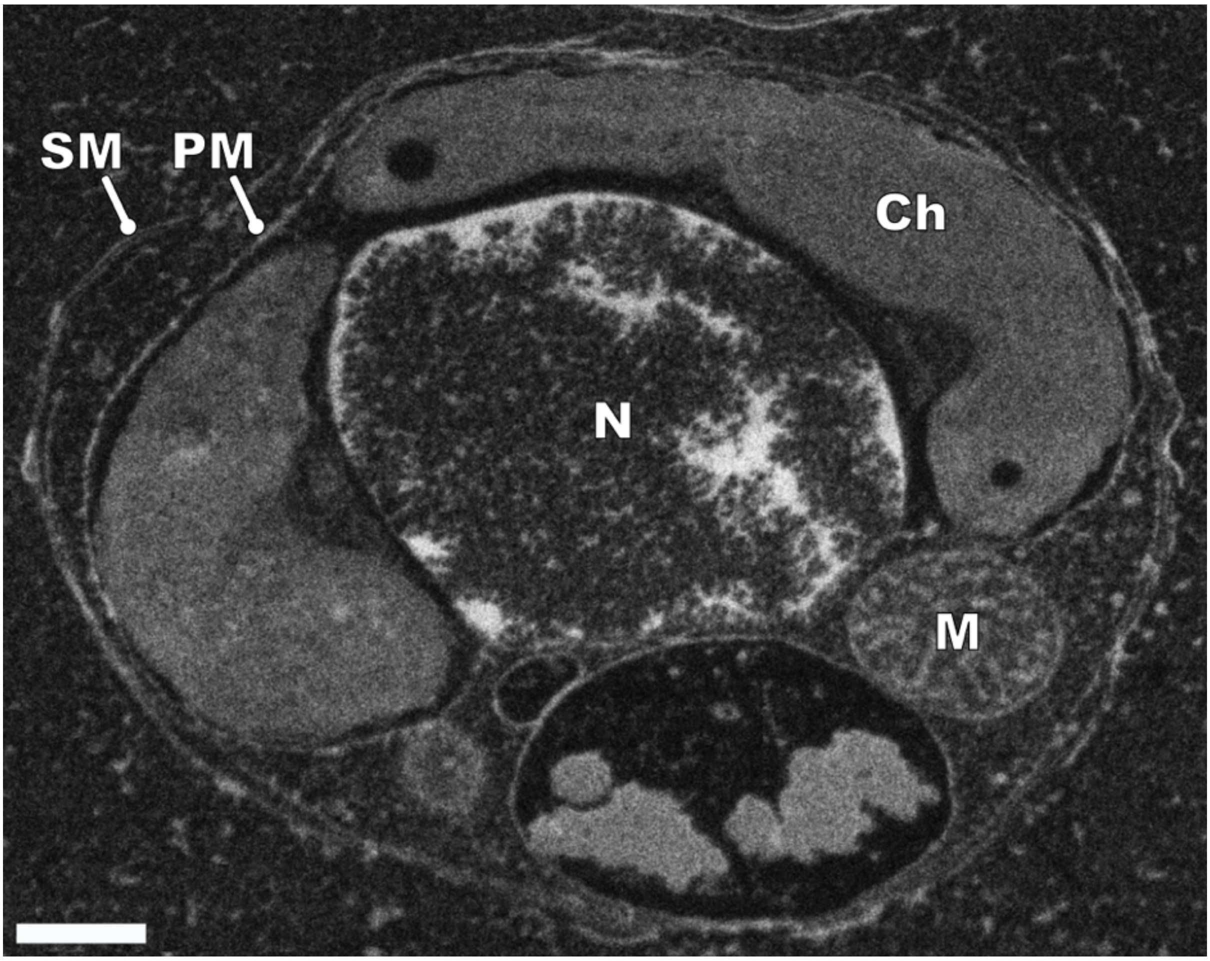


**Fig. S14. Morphology of native *E. huxleyi* cell.** Scanning electron micrograph of a high-pressure vitrified and resin-embedded sample of non-calcifying *E. huxleyi* CCMP 2090 cell. Ch chloroplast, M mitochondrion, N nucleus, SM surface membrane, and PM plasma membrane. Please note that the cell envelope, which covers most *E. huxleyi* cells when imaged using cryo-electron microscopy (Fig. 3), is not resolved in the resin-embedded samples, probably because it was dissolved during the sample preparation procedure or not stained by osmium tetroxide and uranyl acetate used for sample contrasting. Scale bar 500 nm.

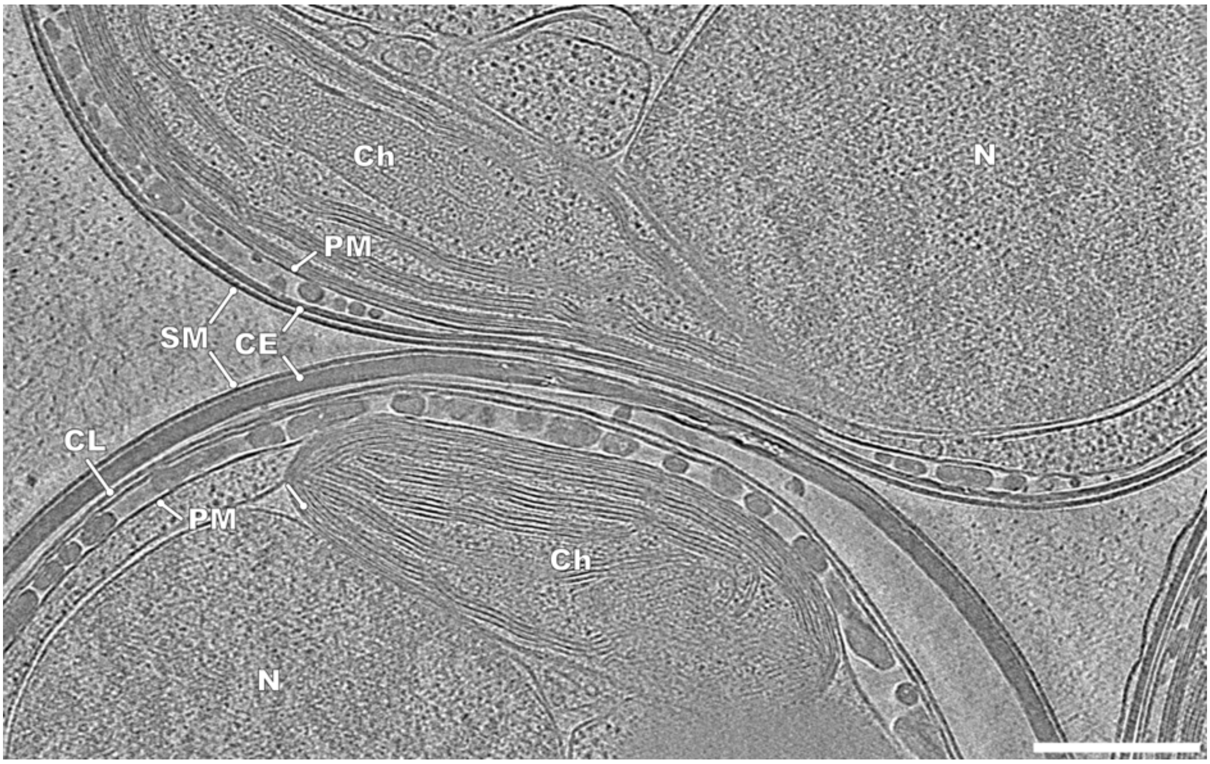


**Fig. S15. Heterogeneity in the thickness of *E. huxleyi* cell envelope.** A projection image of a 30-nm-thick tomogram section of a control non-infected *E. huxleyi* cells with a thick cell envelope (CE) – bottom cell, and a thin cell envelope – upper cell, surface membrane (SM), Ch chloroplast, CL cytoplasmic leaflet, N nucleus, PM plasma membrane**.** Scale bar 200 nm.

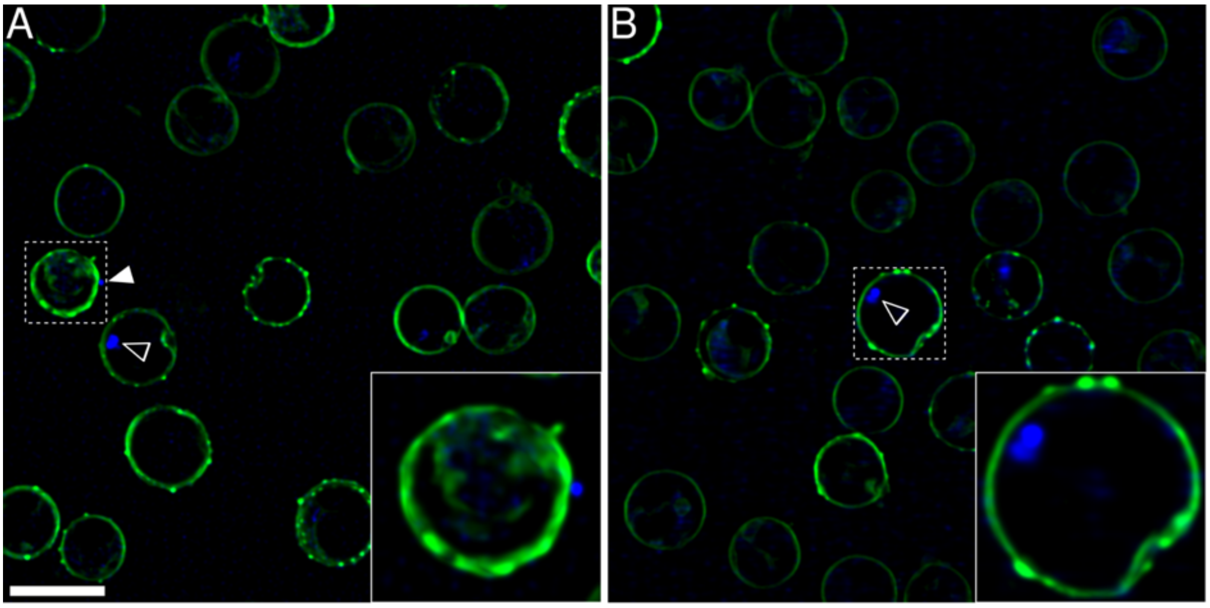


**Fig. S16. EhV-201 attachment to *E. huxleyi* cells. (A, B)** Maximum intensity projections of 2.8-µm-thick volumes of fluorescence confocal sections showing plasma membrane of *E. huxleyi* cells in green (stained with FM 1-43) and EhV-201 in blue (stained with DAPI). (A) *E. huxleyi* cells infected at MOI 100. The EhV-201 particle attached to the cell surface is indicated by a white arrowhead. The inset shows details of the cell with a virus attached from the outside. (B) Non-infected control cells. Many *E. huxleyi* cells contain pigment granules that produce a blue signal (indicated by a black arrowhead with a white outline). However, the pigment granules were never observed to be attached to a cell surface. The inset shows detail of the fluorescent granule inside a control cell. Scale bar 5 µm.

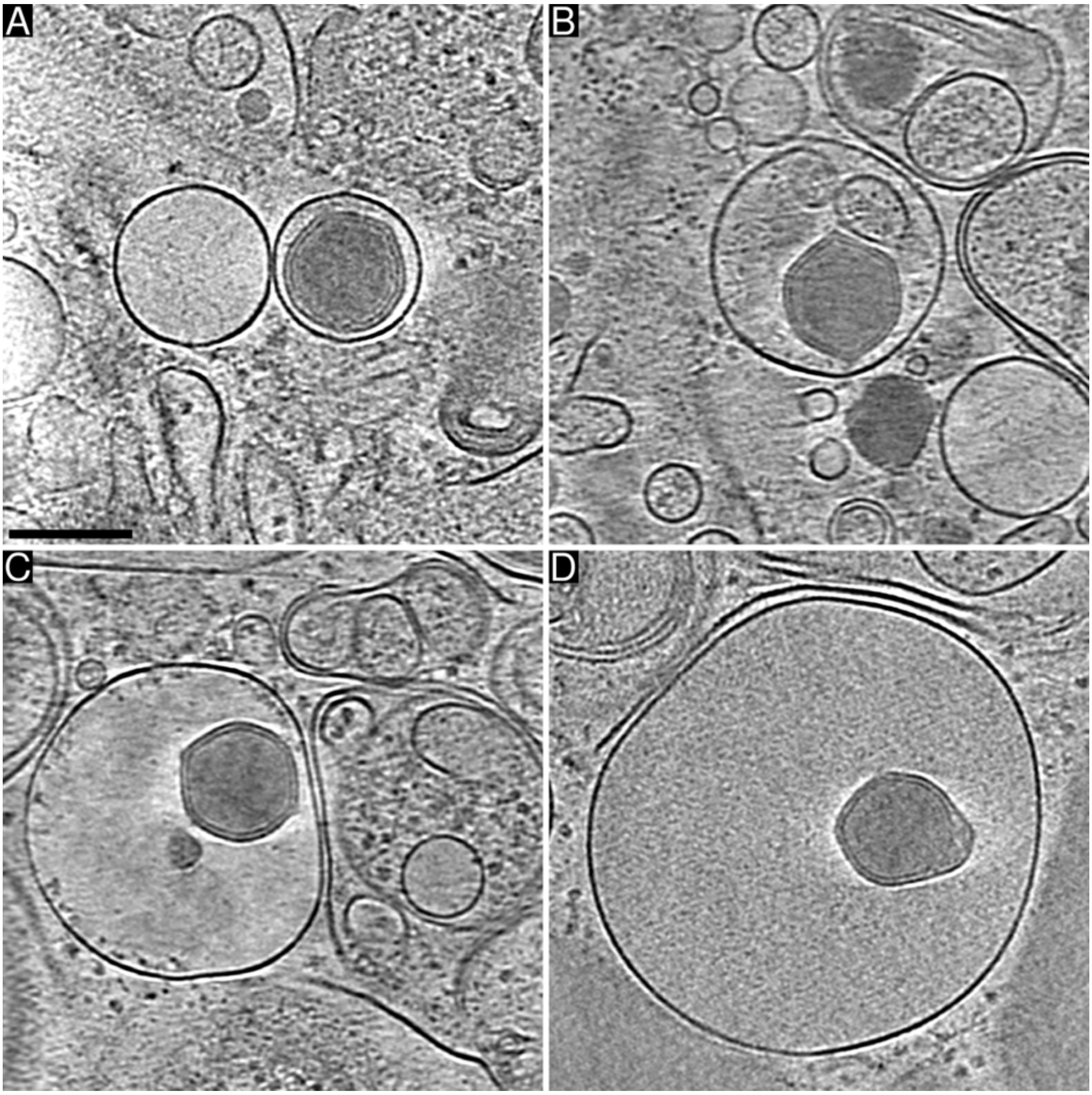


**Fig. S17. Lysis of infected *E. huxleyi* cells results in release of EhV-201 virions inside vesicles. (A-D)** Projection images of 30-nm-thick tomogram sections of vesicles released from a lysed *E. huxleyi* cell. Scale bar 200 nm.

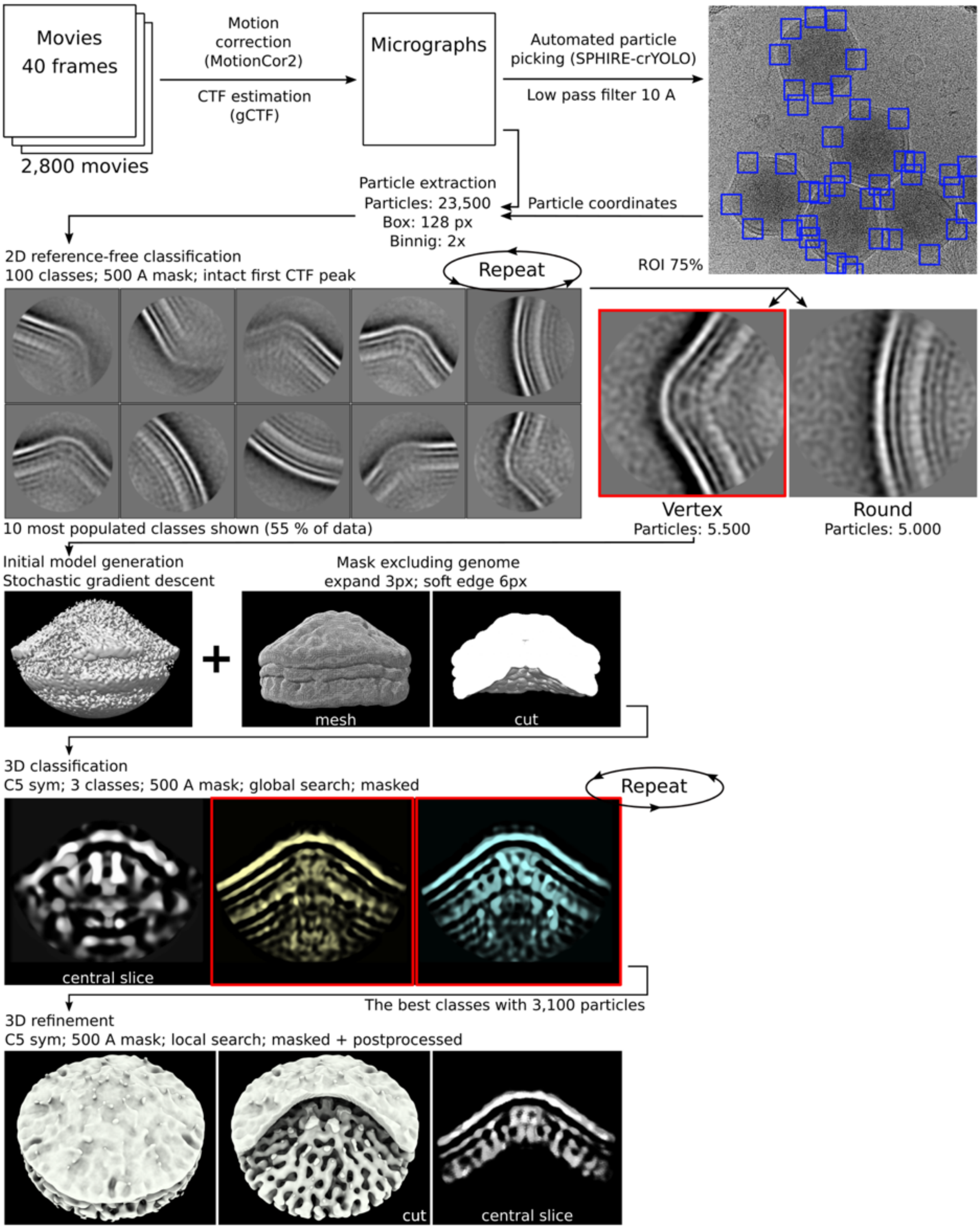


**Fig. S18. Scheme of single-particle reconstruction of EhV-201 virion vertices.**

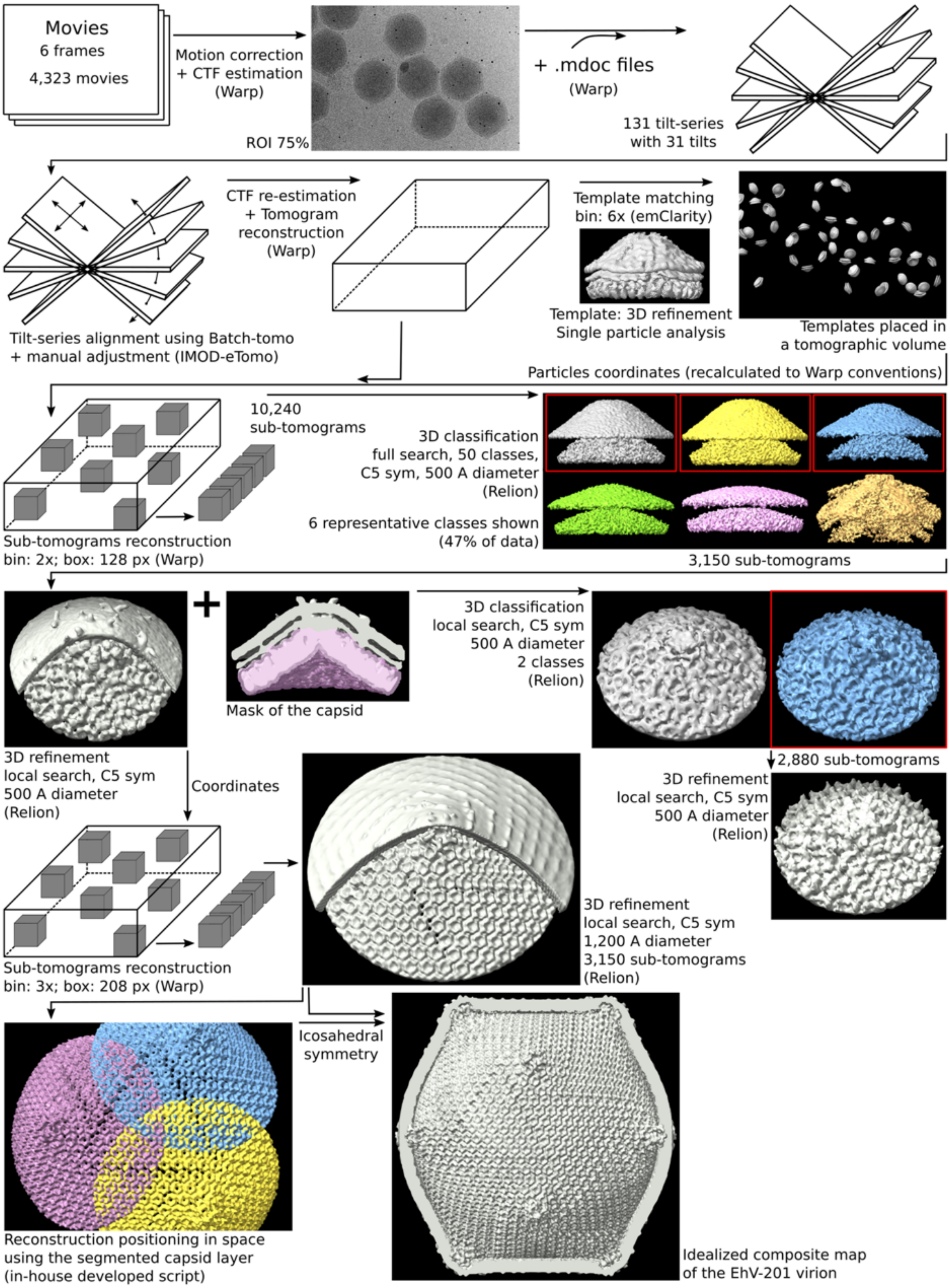


**Fig. S19. Scheme of sub-tomogram reconstruction of EhV-201 virion vertices.**

**Supplementary movies:**

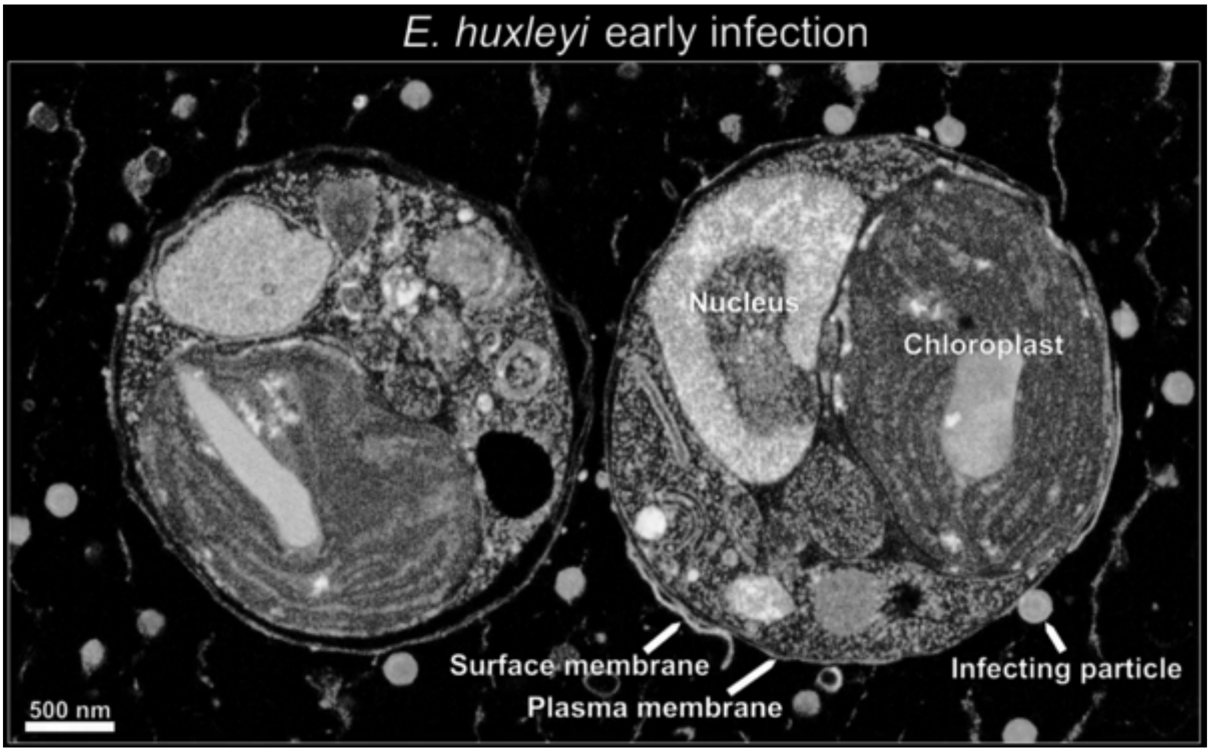


**Movie S1: Attachment and genome delivery of EhV-201.** The movie shows a sequence of scanning electron micrographs of high-pressure vitrified and resin-embedded *E. huxleyi* cells infected by EhV-201 at MOI = 10, 30 min post-infection.

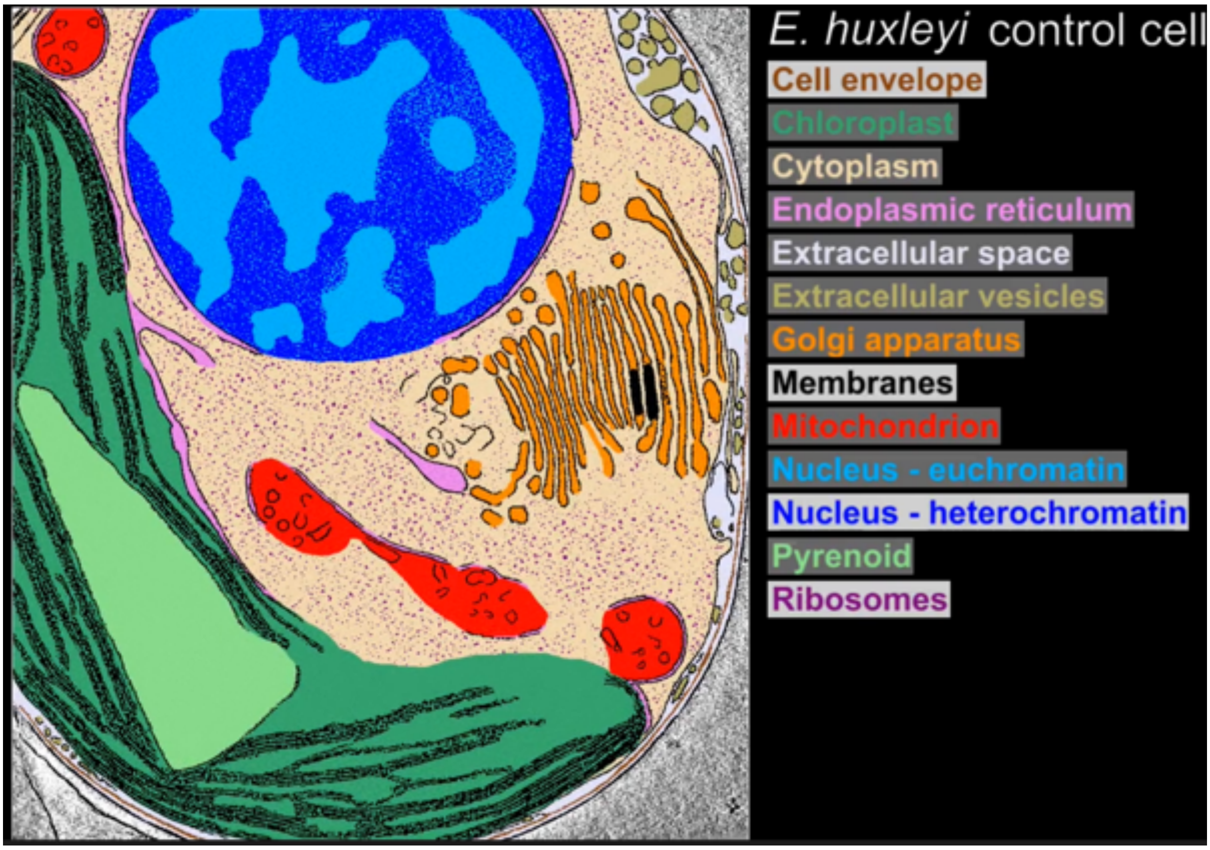


**Movie S2: Native structure of *E. huxleyi* cell**. The movie shows a series of projection images from a cryo-tomogram of a native *E. huxleyi* cell from the non-calcifying strain CCMP 2090. Scale bar 500 nm. The selected slice is segmented and colored according to organelle type.

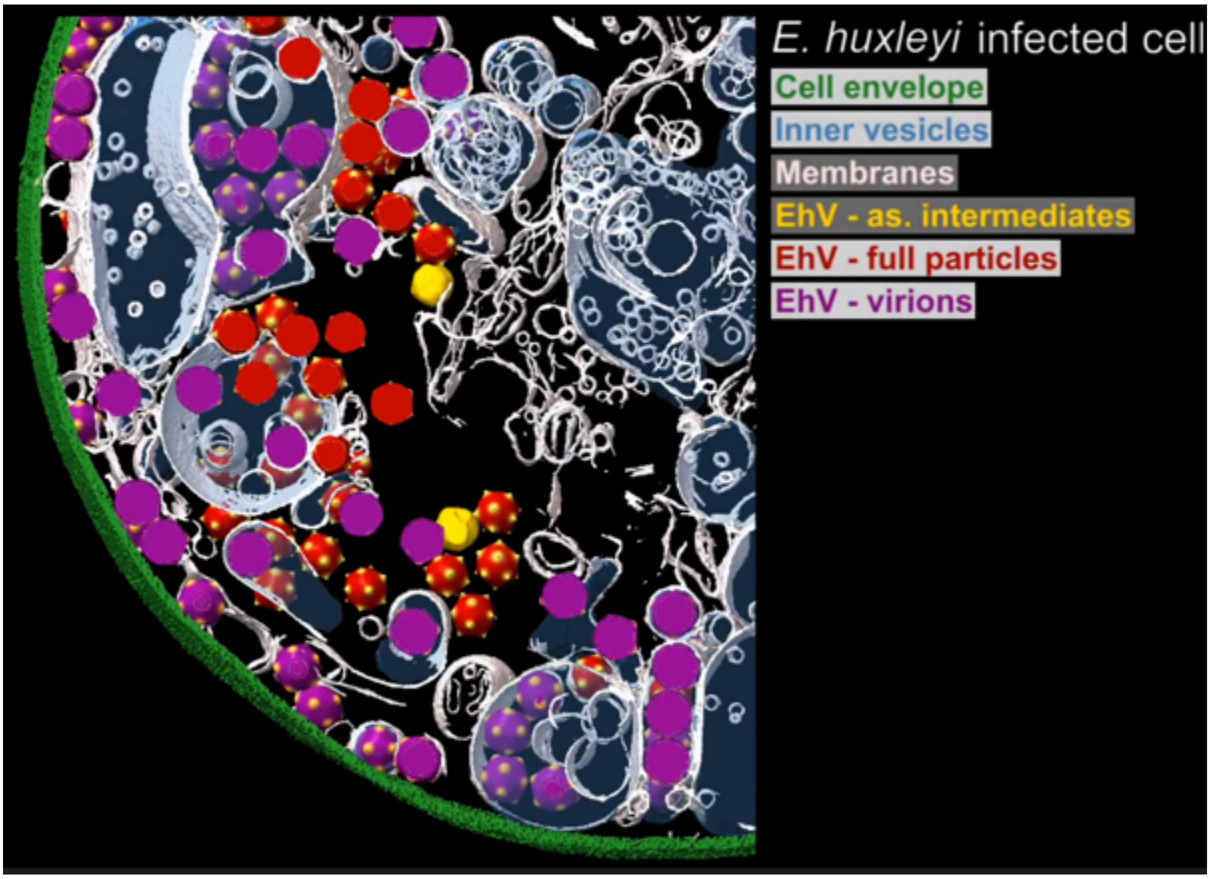


**Movie S3: Structure of EhV-201 replication factory.** The movie shows a series of projection images and a three-dimensional surface representation of a cryo-tomogram of an EhV-201-infected cell. The cell envelope is shown in green, cellular membranes in white, the content of intracellular vesicles is highlighted with semi-transparent blue, virions in red, full particles in orange, and assembly intermediates in yellow. Scale bar 500 nm.

**Supplementary tables:**

**Table S1. Cryo-EM data acquisition parameters, image processing statistics, and structure quality indicators**

| **Cryo-EM data collection** | | | | |
|---|---|---|---|---|
| Data dedicated for | **Single particle analysis** | **Subtomogram averaging** | | **Lamella tomography** |
| *Microscope settings* | | | | |
| Microscope | Titan Krios G2 | Titan Krios G2 | | Titan Krios G2 |
| Voltage (kV) | 300 | 300 | | 300 |
| Projection mode | TEM | EFTEM | | EFTEM |
| Magnification | 37 k | 42 k | | 11,5 k to 19,5 k |
| Cs (mm) | 2,70 | 2,70 | | 2,70 |
| Defocus range (step) (-µm) | 1,2 to 2,4 (0,2) | 2,0 to 4,0 (0,2) | | 25 |
| Detector | Falcon 3EC | K3 | | K2 |
| Energy filter | na | Bioquantum | | Quantum |
| Energy filter mode | na | Zero loss | | Zero loss |
| Energy slit width (eV) | na | 10 | | 20 |
| Detector acq. mode | Linear (integration) | Correlated-double sampling (CDS) | | Linear (integration) |
| Pixel size (Å) | 2,27 | 2,08 | | 7,4 to 13 |
| Flux on detector ($e^-$/px/s) | 72 | 7,5 | | 50 (*) |
| Dose ($e^-$/Å$^2$/s) | 14 | 1,61 | | 0,6 |
| Dose ($e^-$/Å$^2$/tilt) | na | 2,42 | | 0,9 |
| Total dose ($e^-$/Å$^2$) | 28 | 80 | | 41 |
| Output data format | MRC | TIFF | | TIFF |
| Number of micrographs | 2800 | 4323 | | na |
| Number of tilt-series | na | 131 | | 100 |
| *Tilt series setting* | | | | |
| Tilt series acquisition mode | na | Dose symmetric | | Dose symmetric |
| Starting tilt (deg) | na | 0 | | 8 (+ or -) |
| Increment step (deg) | na | 3 | | 3 |
| Maximum tilt (deg) | na | 48 (+ and -) | | Start tilt +45 and -45 |
| Tilt scheme example (deg) | na | 0;+3;+6;+9;+12;-3;-6;-9;-12;+15;... | | Start tilt;+3;+6;-3;-6;+9;... |
| Tracking | na | Everytime | | Everytime |
| Focusing | na | Start tilt + after each 4 | | Start tilt |
| **Cryo-EM reconstruction** | | | | |
| Reconstruction method | **Single particle analysis** | **Subtomogram averaging** | | **Tomogram reconstruction** |
| | | **50 nm diameter** | **120 nm diameter** | |
| Initial particle images (no.) | 23500 | 10240 | 3150 | na |
| Particles box | 128 | 128 | 208 | na |
| Binning | 2 | 2 | 3 | 2 to 4 |
| Particle circular mask (Å) | 500 | 500 | 1200 | na |
| Final particle images (no.) | 3100 | 3150 | 3150 | na |
| Symmetry imposed | C5 | C5 | C5 | C1 |
| Map resolution (Å) | 25 | 13 | 18 | 52 to 60 |
| Resolution method | FSC 0,143 (Gold standard) | FSC 0,143 (Gold standard) | | Theoretical Nyquist |
| Reconstruction software | Relion 3.1 | Relion 4.0 | | IMOD (eTomo) |
| Accession number | EMD-17650 | EMD-19036,17649<br>PDB 8RBT | EMD-17651 | na |
| **Remarks:** (*) a' 50 % of electrons absorbed by lamella | | | | |

## Table S2. Structure similarity analysis of EhV-201 penton protein.

| Structural domains of EhV-201 penton protein | | | | | | | |
|---|---|---|---|---|---|---|---|
| Domain | Residue range | Length (aa) | Structural similarity [a] | | | | |
| | | | Z-score | r.m.s.d. (Å) | aligned residues (aa) | PDB hit | Molecule description |
| **J1** | 1-43, 582-663 | 125 | 8.7 | 2.9 | 109 | 6g42-D | mavirus penton jellyroll domain |
| | | | 8.6 | 3.0 | 106 | 2c9f-C | aenovirus penton jellyroll domain |
| | | | 6.9 | 3.1 | 115 | 3j26-N | sputnik virophage penton jellyroll domain |
| **D1** | 44-57, 259-286, 406-426, 534-581 | 111 | 7.1 | 2.9 | 94 | 2c9f-B | adenovirus penton base |
| | | | 5.6 | 3.4 | 93 | 6g42-C | mavirus penton base |
| | | | 5.0 | 2.9 | 80 | 6iwy-A | flagellar hook-associated protein 2 |
| | | | 6.1 | 2.6 | 85 | 8env-A | phage e217 sheath protein insertion domain [b] |
| **D2** | 58-87, 195-258 | 94 | 6.4 | 2.8 | 85 | 6g42-C | mavirus penton base |
| | | | 5.6 | 3.2 | 80 | 5h5w-B | flagellar hook-associated protein 2 |
| | | | 5.3 | 3.3 | 83 | 2c9f-B | adenovirus penton |
| | | | 4.7 | 2.4 | 76 | 8env-A | phage e217 sheath protein insertion domain [b] |
| **D3** | 88-194 | 107 | 7.0 | 2.5 | 68 | 6dc6-C | ubiquitin-like modifier enzyme 1 FCCH domain |
| | | | 3.5 | 3.5 | 63 | 7qof-D | phage capsid protein |
| | | | 3.0 | 3.0 | 69 | 7y22-Y | phage connector protein |
| **D4** | 287-405 | 119 | 7.6 | 3.2 | 108 | 6g42-C | mavirus penton base |
| | | | 7.6 | 2.5 | 99 | 2c9f-B | adenovirus penton |
| | | | 6.1 | 2.6 | 86 | 6iwy-A | flagellar hook-associated protein 2 |
| **D5** | 427-533 | 107 | 5.0 | 2.7 | 74 | 6h9v-A | norovirus P-domain |
| | | | 4.1 | 2.4 | 61 | 8eci-1 | phage decoration protein |
| | | | 2.7 | 3.3 | 68 | 4ruf5-C | phage tail protein insertion domain |

a, DALI PDB100 search hit; b, DALI PDB25 search hit

## Table S3. F/2-Si medium composition

| Alga medium ingredients | | | |
|---|---|---|---|
| **Ingredient** | **Common name / formula** | **Manufacturer** | **Cat.No./Product No./Link** |
| Sea water from a functional marine aquaria | Regenerated sea water | Aqua Vala (Brno, Czech Republic) | shop.akvaristika-morska.cz |
| *Micronutrients* | | | |
| Biotin | Vitamin H or B7 | Sigma Aldrich (Merck) | B4639 |
| Cobalamine | Vitamin B12 | Sigma Aldrich | V6629 |
| Cobalt chloride | $CoCl_2$ | Sigma Aldrich | 255599 |
| Cupric sulfate | $CuSO_4$ | Sigma Aldrich | 209198 |
| Ethylenediaminetetraacetic acid disodium salt | $Na_2EDTA$ | Sigma Aldrich | 324503 |
| Ferric chloride | FeCl3 | Sigma Aldrich | 236489 |
| Manganese chloride | $MnCl_2$ | Sigma Aldrich | 221279 |
| Monosodium phosphate | $NaH_2PO_4$ | Sigma Aldrich | S0751 |
| Sodium molybdate | $Na_2Mo_4$ | Sigma Aldrich | 331058 |
| Sodium nitrate | $NaNO_3$ | Merck (Darmstadt, Germany) | 106537 |
| Thiamin | Vitamin B1 | Sigma Aldrich | T1270 |
| Zinc sulphate | $ZnSO_4$ | Sigma Aldrich | 221376 |

**Table S4. Conditions used to prepare grids with EhV-201 virions for recording data for single-particle reconstruction and tomographic tilt-series and grids with EhV-201-infected *E. huxleyi* cells**

| Cryo-preservation settings | | | |
|---|---|---|---|
| Data dedicated for | **Single particle analysis** | **Subtomogram averaging** | **Lamella preparation** |
| *Grid specification* | | | |
| Grid material | Copper | Copper | Gold |
| Mesh | 300 | 200 | 200 |
| Coating material | Carbon | Carbon | Gold |
| Hole diameter (μm) | 2 | 2 | 2 |
| Hole spacing (μm) | 1 | 1 | 1 |
| Manufacturer | Quantifoil | Quantifoil | Quantifoil |
| *Glow discharge settings* | | | |
| Device | Gatan Solarius | Gatan Solarius | Gatan Solarius |
| Atmosphere | Hydrogen-oxygen | Hydrogen-oxygen | Hydrogen-oxygen |
| Pirani pressure (Pa) | 0,05 | 0,05 | 0,05 |
| Power (W) | 40 | 40 | 40 |
| Time (s) | 15 | 15 | 15 |
| Glow discharged side | Coating side up | Coating side up | Coating side up |
| *Chamber conditions* | | | |
| Vitrification device | Vitrobot Mark IV | Vitrobot Mark IV | Vitrobot Mark IV |
| Humidity (%) | 100 | 100 | 100 |
| Temperature (°C) | 10 | 10 | 20 |
| Blotting time (s) | 3 | 3 | 12 |
| Blotting force | -2 | -2 | -2 |
| Wait time (s) | 10 | 10 | 10 |
| Blotting paper - front | Filter paper | Filter paper | Wax-soaked filter paper |
| Blotting paper - back | Filter paper | Filter paper | Filter paper |
| Drain time (s) | 0 | 0 | 0 |
| Sample volume (μl) | 3,5 | 3,5 | 4 |
| Vitrification medium | Ethane | Ethane | Ethane |
| Storage medium | Nitrogen | Nitrogen | Nitrogen |

**Supplementary files:**

**Supplementary file 1. Multiple sequence alignment of penton proteins from NCVs used for PSI-BLAST identification of EhV penton protein.**
